# Targeted depletion of *pks*+ bacteria from a fecal microbiota using specific antibodies

**DOI:** 10.1128/msystems.00079-23

**Published:** 2023-05-23

**Authors:** Aitor Blanco-Míguez, Raquel Marcos-Fernández, Lucía Guadamuro-García, Florentino Fdez-Riverola, Joaquín Cubiella, Anália Lourenço, Abelardo Margolles, Borja Sánchez

**Affiliations:** 1 Department of Microbiology and Biochemistry of Dairy Products, Instituto de Productos Lácteos de Asturias (IPLA), Consejo Superior de Investigaciones Científicas (CSIC), Villaviciosa, Asturias, Spain; 2 ESEI: Escuela Superior de Ingeniería Informática, University of Vigo, Edificio Politécnico, Campus Universitario As Lagoas, Ourense, Spain; 3 CINBIO - Centro de Investigaciones Biomédicas, University of Vigo, Campus Universitario Lagoas-Marcosende, Vigo, Spain; 4 SING Research Group, Galicia Sur Health Research Institute (IIS Galicia Sur), SERGAS-UVIGO, Hospital Álvaro Cunqueiro, Vigo, Spain; 5 Department of Gastroenterology, Complexo Hospitalario Universitario de Ourense, Instituto de Investigación Sanitaria Galicia Sur, CIBEREHD, Ourense, Spain; 6 CEB - Centre of Biological Engineering, University of Minho, Campus de Gualtar, Braga, Portugal; 7 Functionality and Ecology of Beneficial Microbes (MicroHealth) Group, Instituto de Investigación Sanitaria del Principado de Asturias (ISPA), Oviedo, Asturias, Spain; NIAID, NIH, Bethesda, MD, USA

**Keywords:** *pks* island, colibactin, *Escherichia coli*, flow cytometry

## Abstract

The *pks* island is one of the most prevalent pathogenicity islands among the *Escherichia coli* strains that colonize the colon of colorectal carcinoma (CRC) patients. This pathogenic island encodes the production of a nonribosomal polyketide-peptide named colibactin, which induces double-strand breaks in DNA molecules. Detection or even depletion of this *pks*-producing bacteria could help to understand the role of these strains in the context of CRC. In this work, we performed a large-scale *in silico* screening of the *pks* cluster in more than 6,000 isolates of *E. coli*. The results obtained reveal that not all the *pks*-detected strains could produce a functional genotoxin and, using antibodies against *pks*-specific peptides from surface cell proteins, a methodology for detection and depletion of *pks*+ bacteria in gut microbiotas was proposed. With our method, we were able to deplete a human gut microbiota of this *pks*+ strains, opening the door to strain-directed microbiota modification and intervention studies that allow us to understand the relation between these genotoxic strains and some gastrointestinal diseases.

**IMPORTANCE:**

The human gut microbiome has also been hypothesized to play a crucial role in the development and progression of colorectal carcinoma (CRC). Between the microorganisms of this community, the *Escherichia coli* strains carrying the *pks* genomic island were shown to be capable of promoting colon tumorigenesis in a colorectal cancer mouse model, and their presence seems to be directly related to a distinct mutational signature in patients suffering CRC. This work proposes a novel method for the detection and depletion of *pks*-carrying bacteria in human gut microbiotas. In contrast to methods based on probes, this methodology allows the depletion of low-abundance bacterial strains maintaining the viability of both targeted and non-targeted fractions of the microbiota, allowing the study of the contribution of these *pks*-carrying strains to different diseases, such as CRC, and their role in other physiological, metabolic or immune processes.

## INTRODUCTION

The *pks* island is one of the most prevalent pathogenicity islands among the *Escherichia coli* strains that colonize the colon of colorectal carcinoma (CRC) patients (1). This genomic island codes for multienzymatic biosynthesis machinery, including nonribosomal peptide synthetases (NRPSs), polyketide synthases (PKSs), an efflux pump, and additional enzymes that allow the synthesis of a peptide-polyketide hybrid genotoxin, named colibactin (2, 3). Colibactin is known to induce double-strand breaks in the DNA, chromosome aberrations, cell cycle arrest in G2/*M* phase and increased lymphopenia in septic rodents (2, 4 - 7). For its biosynthesis, the NRPSs and PKSs are modified post-translationally allowing the acceptance of synthesis building blocks by the ClbA phosphopantetheinyl transferase, that is, acetyl-, malonyl-, or methylmalonyl-CoA monomers for PKS or amino acid monomers; and proteinogenic or non-proteinogenic amino acids for NRPS (8, 9). The NRPSs and PKSs then function as a multimodular assembly line that synthesizes an inactive precolibactin. Precolibactin biosynthetic intermediates are offloaded from the assembly line by the ClbQ thioesterase, thus possibly regulating colibactin synthesis and genotoxic activity (10, 11). Once precolibactin synthesis is finished, the prodrug is taken in charge by ClbM, a multidrug and toxic compound extrusion (MATE) transporter, and released into the periplasmic space (12). Once into the periplasmic space, precolibactin is matured by the ClbP peptidase, which will generate the mature colibactin (13, 14) via removal of the *N*-myristoyl-d-Asn side chain (15, 16). However, the mechanism by which colibactin is exported outside the bacteria is still unexplained (17). As a supplemental self-protection mechanism, the *pks* island encodes the ClbS resistance protein, a cyclopropane hydrolase that inactivates colibactin in the producing bacteria (18, 19).

The *pks* island was detected predominantly in *E. coli* strains of phylogenetic group B2 (2, 4, 20), and the prevalence of these strains has increased significantly in developed countries (21, 22). Interestingly, *E. coli* strains carrying the *pks* island (*pks*+ strains) are present in about 20% of healthy individuals, about 40% of patients with inflammatory bowel disease, and about 60% of patients with familial adenomatous polyposis or CRC (23 - 27). Moreover, one such commensal, the probiotic strain Nissle 1917, commercialized as Mutaflor and efficacy and safety in maintaining remission of intestinal inflammation (28), contains the *pks* island and produces a functional genotoxin (2). Several studies have also found this pathogenic island in other members of the *Enterobacteriaceae* family, such as *Citrobacter*, *Klebsiella*, and *Enterobacter* genus (29), in commensal species, such as *Frischella perrara* (30), *Pseudovibrio* (31) and, more recently, in other families from the Enterobacteriales order (32), suggesting that its transmission is carried out via horizontal gene transfer (29, 33). Whether harboring *pks*+ strains in the long term is a risk factor for developing colorectal tumors is left for further epidemiological studies. However, it has already been reported a distinct mutational signature in patients suffering CRC that seem to be directly related to the exposure to bacteria carrying the *pks* pathogenicity island (34).

Flow cytometry (FC) has been used for the analysis of bacteria since the 1970s (35 - 37). FC allows individual cell analysis and presents some advantages such as rapid data acquisition and multiparameter analysis. There are a lot of applications for FC in the field of microbiology (38, 39), and recent studies have used this technique for the detection, isolation, and cultivation of specific bacteria targeting predicted cell surface proteins (40, 41).

In the present article, we developed an *in silico* method for the detection of the *pks* island and we applied it on more than 6,000 *E. coli* human isolates from the PATRIC database. Results showed that several genes from this cluster are exclusive of these colibactin-producing strains, and we discovered *pks*-specific peptides derived from these *pks*-associated genes. Using antibodies against four of these *pks*-specific peptides, an experimental methodology for detection and depletion of *pks*+ bacteria in gut microbiotas was proposed. This methodology can be applied for the modification of a microbiota in a target manner, allowing the study of the contribution of these *pks*-carrying strains to different diseases, such as CRC, or their role in other physiological, metabolic, and immune processes. The main results are presented next.

## MATERIALS AND METHODS

### Bacterial genomes and proteomes

The proteome sequence data of the *E. coli* strains considered in this study were downloaded from the PATRIC database (42). In total, 6,212 strains isolated from humans (tagged *Homo sapiens* at PATRIC) were retrieved from their public FTP site (ftp://ftp.patricbrc.org/genomes/). Accession numbers are listed in Table S1.

### Distribution of the colibactin genome island

In total, 6,212 *E. coli* strains isolated from humans and deposited in the PATRIC database were selected for the study of the distribution and structure of the colibactin genome island (*pks*+ strains). For colibactin island detection, proteomes from the different strains were aligned against the published sequence of the colibactin genome island of the *E. coli* strain IHE3034 (accession no. AM229678) using BLASTp. For positive detection, 20 out of 23 genes must be detected with less than five gaps between consecutive genes. For each gene, a cutoff value was set based on the alignment expected value (*E*-value < 1e^−5^), sequence coverage (coverage > 80%), and identity (identity > 80%). The complete list of *pks*+ strains is listed in Table S1.

### Selection and synthesis of *pks*+ unique peptides

The proteins of the colibactin genomic island of the *E. coli* strain IHE3034 were split into peptides of 20 amino acids. Later, the proteomes of the 6,212 *E. coli* strains from the PATRIC database were aligned against these peptides using BLASTp. For positive detection, a cutoff value was set based on the alignment expected value (*E*-value < 1e^−5^), sequence coverage (coverage > 80%), and identity (identity > 80%). The four peptides with the biggest detection score on the *pks*+ strains and the lowest detection score on the *pks*− strains were selected. These 20 amino acid length peptides were located, according to PSORTb v3.0 subcellular predictions (43), on periplasmic loops of three membrane proteins, that is, clbH (NRPS), clbC (PKS), and clbD (NRPS/PKS).

The selected four peptides were further aligned against the NCBI RefSeq database (accessed at 16 March 2023) (44) to screen for positive hits against other bacterial taxa. For positive detection, a cutoff value was set based on the alignment expected value (*E*-value < 1e^−5^), sequence coverage (coverage > 80%), and identity (identity > 80%). Non-*E*. *coli* positive hits are reported in Table S3.

About 5 mg of the four *pks*-specific peptides was synthesized at the Genecust Europe facilities (Laboratoire de Biotechnologie du Luxemburg S.A., Dudelange, Luxembourg), with a minimum purity of 95%. Peptides were conjugated to the keyhole limpet hemocyanin as carrier protein to favor the generation of peptide-specific antibodies.

### Control bacterial strains and growth conditions

*E. coli* LMG2092 and Nissle 1917 strains were selected as negative and positive control for *pk*s+ bacteria, respectively. Both strains were grown in Luria-Bertani (LB) broth containing 10 g/L tryptone (Biokar Diagnostics, France), 5 g/L yeast extract (Biokar Diagnostics, France), 10 g/L of NaCl (Merck, KGaA, Darmstadt, Germany), and 1 L of deionized H_2_O. All cultures were grown on the surface of agar plates at 37°C in an MG500 anaerobic chamber (Don Whitley Scientific, West Yorkshire 100, UK) with an atmosphere of 10% (v/v) H_2_, 10% CO_2_, and 80% N_2_ for 48 h. After that, 1 single colony was inoculated in 4 mL of fresh liquid media and all cultures were incubated at 37°C for 24 h in anaerobic, aerobic, and aerobic with shaking conditions. The next day, 100 µL of the bacteria suspension was inoculated into 4 mL of fresh medium and incubated at 37°C until an optical density (OD_600_) of about 0.6 after approximately 3 h. After that, cultures were harvested by centrifugation, washed once with bacterial FC buffer (Miltenyi, Bergisch Gladbach, Germany) and resuspended in the same buffer to an OD_600_ = 0.2, which represents around 1 × 10^8^ CFUs/mL.

The bacteria were also grown in fresh liquid media of Nutrient broth (Oxoid, Ltd., Basingstoke, Hampshire, UK) and nutrient broth supplemented with 2% of glucose (Sigma-Aldrich, St. Louis, MO, USA).

### Fecal sample collection and microbiota extraction

The study sample comprised of 13 fecal samples from healthy donors. Fresh fecal material from healthy donors was collected in a sterile container and immediately manipulated and homogenized within a maximum of 2 h from defecation. About 9 mL of sterile NaCl 0.9% (w/v) was added to 1 g of the sample, and the mixture was homogenized in a sterile bag using a laboratory paddle blender (Stomacher Lab Blender 400, Seward Ltd., UK). Microbiota extraction was then performed following the protocol described by Hevia et al. (45). A solution of Nycodenz 80% (w/v) (PROGEN Biotechnik GmbH, Heidelberg, Denmark) was prepared in ultrapure water, and sterilized at 121°C for 15  min. A volume of 3  mL of the diluted, homogenized fecal sample was placed on top of 1 mL of the Nycodenz solution and centrifuged for 40  min at 4°C (9,000*g*, TST41.14 rotor, Kontron, Milan, Italy). The upper phase (soluble debris) was discarded after centrifugation, and the layer corresponding to the microbiota was collected, washed once, and resuspended in 1 mL of FC buffer (1× MACSQuant Running Buffer, Miltenyi Biotec, Germany).

### Polyclonal antibody generation

Polyclonal sera against the purified *pks*-specific peptides were generated in the Central Facilities of the University of Oviedo (Spain). A rabbit was immunized five times, with an interval of 15 days between immunizations, with 500 µg of peptide dissolved in 1 mL of phosphate-buffered saline (PBS) and mixed with 1 mL of Freund’s Incomplete Adjuvant. The rabbit was finally sacrificed by intracardiac puncture and blood was let to coagulate at 37°C for 4 h and subsequent overnight incubation at 4°C. Serum was separated by centrifugation (30 min, 2,000*g*), and used for purifying the IgG. First, ammonium sulfate was added to a final concentration of 45% (w/v), and the mix was incubated overnight at 4°C. After centrifugation (1 h, 10,000*g*, 4°C), the pellet was resuspended in 30 mL of PBS. This was extensively dialysed against PBS, and loaded in a ProteinA Sepharose 4 Fast Flow, previously equilibrated with 10 column volumes of PBS (50 mL). The column was washed with six column volumes of PBS, and five fractions of 5 mL were eluted with citric acid 100 mM pH 3.0. pH was corrected in each aliquot by adding 1 mL of 1M Tris-HCl pH 9.0. Fractions were mixed, centrifuged in a Vivaspin 20 device (3,000*g*, molecular weight cutoff of 10 kDa) and washed with 20 mL of PBS. Protein concentration was estimated by measuring the *A*_280_ and samples were aliquoted and stored at −80°C.

### Antibody conjugation

Polyclonal of antipeptide 1, antipeptide 2, antipeptide 3, and antipeptide 4 serum IgG fractions were conjugated with fluorescein isothiocyanate (FITC) and allophycocyanin (APC) using the commercial kits (Abcam, Cambridge, MA, USA) and following the manufacturer’s instructions. For FITC and APC conjugation, the antipeptide polyclonal antibodies were reconstituted in amine-free PBS. About 100 mL of each 1.5 mg/mL antibody was added to the reactive dye for each conjugation. Before adding the antibody to the FITC/APC mix, 10 µL of FITC-Modifier or APC-Modifier reagent was added to the antibody. The antibody-dye mixtures were incubated in the dark at room temperature (RT; 20–25°C) for 3 h. After incubation, 10 µL of FITC-Quencher or APC-Quencher reagent was added and gently mixed. The concentration of antibodies in the final sample was 20 µg/mL.

### FC analysis

Labeled cells with polyclonal antibodies were acquired and analyzed in a MACSQuant Flow Cytometer device (Miltenyi Biotec, Germany) using the following acquisition parameters: flow rate set to “low,” uptake volume of 10 µL, FSC set to hyperlogarithmic amplification (370 V), SSC set to hyperlogarithmic amplification (440 V), channel B1 corresponding to the FITC detection set to hyperlogarithmic amplification (370 V) and channel R1 corresponding to the APC detection set to hyperlogarithmic amplification (360 V). At least 10,000 events were acquired in each run.

### Detection of *E. coli* Nissle 1917 and LMG2092 by FC

The binding capability of the four antipeptides antibodies was tested over a *pks*+ (*E. coli* Nissle 1977) and a *pks*− (*E. coli* LMG2092) strains.

About 25 µL of the bacterial suspension was mixed with 25 µL of the FITC-conjugated antibody at a final concentration of 20 µg/mL. The samples were incubated for 15 min at RT and were then washed with FC buffer at 13,000 rpm for 5 min. Finally, bacteria were resuspended in 150 µL of FC buffer and samples were acquired using a MACSQuant Flow Cytometry (Miltenyi Biotec, Germany). Bacteria were labeled and analyzed at a different optical density (i.e., OD_600_=0.3, 0.6, 1, and 1.5) and with a different medium (i.e., LB, Nutrient Broth, and Nutrient Broth supplemented with 2% of glucose).

### Detection and depletion of *pks*+ bacteria from gut microbiotas

The detection and depletion of *pks*+ strains were investigated over 13 gut microbiotas from healthy donors. Microbiotas were labeled with antipeptide 2 polyclonal antibodies conjugated to APC and incubated for 15 min at RT. Then, they were washed with FC buffer at 13,000 rpm for 5 min and the supernatant was removed. Finally, microbiotas were resuspended in 150 µL of FC buffer and data were acquired and analyzed using a MACSQuant flow cytometer as indicated previously.

For depletion, microbiotas were labeled with the polyclonal antipeptide 2 serum IgG fraction conjugated to APC and incubated for 15 min at RT. Then, they were washed with FC buffer at 13,000 rpm for 5 min and the supernatant was removed. For the positive selection of *E. coli pks+*, microbiotas were resuspended in 100 µL of resuspension buffer (PBS supplemented with 2 mM EDTA and 3% (v/v) of de-complemented bovine fetal serum). To this mix, 20 µL of magnetic anti-APC microbeads were added (BD Biosciences, San José, CA, USA). The mixture was incubated for 15 min at RT with shaking. Then, the microbiotas were washed at 13,000 rpm for 5 min with 1× BD IMag buffer (BD Biosciences, San José, CA, USA) and the column was conditioned with 1× BD IMag buffer (BD Biosciences, San José, CA, USA). The positive fraction was retained in the magnetic column after three washes with 1× BD IMag buffer (BD Biosciences). Finally, the positive fraction was eluted through the 1× BD IMag buffer. Both positive and negative fractions were further analyzed by FC using a MACSQuant flow cytometer as previously indicated.

### Immunofluorescence microscopy

The binding ability of the antipeptide 1, 2, and 3 antibodies contained in the polyclonal serum was also determined by a confocal scanning laser microscope equipped with a Leica DFC365FX digital camera (DMi8, Leica Microsystems). The bacterial species were grown until exponential phase OD_600_ of 0.7, were then washed with FC buffer, fixated with paraformaldehyde 4%, permeabilized with FC buffer supplemented with 2% of Tween 20 and incubated for 15 min with the polyclonal antibody antipeptides conjugated with FITC (200 µg/mL). Finally, bacteria were washed once with FC buffer, resuspended in 10 µL of flow cytometer buffer and were analyzed with confocal microscopy using a 100× oil objective. The images were acquired with software LasX (Leica Microsystems). The FITC filter cube (excitation 480/40 and emission 527/30) was used.

### Evaluation of the *pks*+ detection and depletion method by qualitative PCR

Conventional qualitative PCR was performed to evaluate the proposed method on four human gut microbiotas from healthy subjects. The four samples were supplemented with 0.1%, 1%, and 10% of *E. coli* Nissle 1917 cells, and the positive and negative fractions were assessed. Primers amplifying a region of the clbB gene were used following previously published protocols and primers (forward primer 5′-GATTTGGATACTGGCGATAACCG-3′ and reverse primer 5′-CCATTTCCGTTTGAGCACAC-3′) (4). About 20 mL of reaction mixture for conventional qualitative PCR consisted of 1 µL of DNA (DNA concentrations are summarized in Table S5), each 0.6 µL of 10 µmol/L forward and reverse primer solutions, deoxynucleotide mixture, the DNA polymerase Master Mix RED (Ampliqon), and H_2_O. The PCR conditions were 10 min at 95°C, 30 cycles of 45 s at 95°C, 45 s at 54°C, and 1 min at 72°C, and a final step of 7 min at 72°C. *E. coli* Nissle 1917 was selected as a positive control for *pks*+ detection.

One of the experiments that was carried out consisted of passing a mixture of *E. coli* Nissle 1917 and *E. coli* LMG2092 labeled with polyclonal antibody antipeptide 2 several times through the magnetic column in order to check if we were able to increase the recovery of *E. coli* Nissle and decrease by the positive fraction the recovery of *E. coli* LMG2092. The mixture contained 50% Nissle and 50% LMG2092. About 250 mL of magnetic beads was added and it was tried to pass one, three, and six times through the column. Results for checking enrichment and depletion of Nissle 1917 and LMG2092 were acquired by FC.

### Isolation and identification of the *pks*+ bacteria

The positive fractions were streaked in agar plates of LB and EC with reduced bile salt (Oxoid) and incubated at 37°C. Random colonies were isolated and grown in fresh liquid LB broth and incubated at 37°C. DNA from isolates was extracted using the GenElute Bacterial Genomic DNA kit (Sigma-Aldrich) and subjected to PCR amplification with primers 27f (5′-AGAGTTTGATCMTGGCTCAG) and 1492r (5′-TACCTTGTTACGACTT). 16S identification was performed, and sequences were aligned against the NCBI nonredundant database using BLAST (46).

### Validation of the *pks*+ isolates by qualitative PCR analysis

Conventional qualitative PCR was performed to evaluate the isolates of the fecal samples retrieved in the positive fraction of the magnetic separation. To detect *pks*+ bacterial DNA, primers amplifying a region of the *clbB* gene were used following the previously published protocols and primers (forward primer 5′-GATTTGGATACTGGCGATAACCG-3′ and reverse primer 5′-CCATTTCCCGTTTGAGCACAC-3′) (2, 47). About 20 mL of reaction mixture for conventional qualitative PCR consisted of 1 µL of DNA (DNA concentrations are summarized in Table S5), each 0.6 µL of 10 µmol/L forward and reverse primer solutions, deoxynucleotide mixture, the DNA polymerase Master Mix RED (Ampliqon), and H_2_O. The PCR conditions were 10 min at 95°C, 30 cycles of 45 s at 95°C, 45 s at 54°C, and 1 min at 72°C, and a final step of 7 min at 72°C. *E. coli* Nissle 1917 was selected as the positive control for *pks*+ detection and *E. coli* LMG2092 as the negative control.

### Genome sequencing, assembly, and annotation

Fourteen isolates from the positive fraction of the fecal samples were selected to sequence their entire genome. DNA library preparation was performed using the Nextera XT DNA sample preparation kit (Illumina, San Diego, CA, USA) according to the manufacturer’s instructions. About 1 ng input DNA from each sample was used for library preparation. The isolated DNA underwent fragmentation, adapter ligation, and amplification. The ready-to-go libraries were pooled equimolarity, denatured and diluted to a sequencing concentration of 2 pM. Sequencing was performed on NextSeq 550 instruments (Illumina, San Diego, CA, USA), according to the manufacturer’s instructions, using the 2 × 150 bp High Output sequencing kit, and spike-in of 1% PhiX control library. Raw sequence reads were preprocessed to remove bad quality and sort reads and assembled using Spades 3.13.0 (48). Contigs with less than 1,000 nt were discarded. The quality of the assembled genomes was checked using CheckM 1.1.2 (49) and sequentially annotated using Prokka 1.12 (50). The assembled genomes are available at https://www.ncbi.nlm.nih.gov/bioproject/PRJNA668898.

### Genome classification and *pks* cluster detection

The assembled genomes were taxonomically classified using two independent approaches. On the one hand, the 16S sequences were retrieved from the assembled genomes using barrnap 0.9 (https://github.com/tseemann/barrnap) and analyzed with RDP classifier (51). On the other hand, the assembled genomes were processed with the phylophlan_metagenomic script of PhyloPhlAn 3.0 (52) over the SGB Jan19 database (53). Moreover, the phylogenetic group of the *E. coli* strains was predicted using EzClermont 0.4.5 (54).

For *pks* island detection, the annotated genomes were aligned against the published sequence of the colibactin genome island of the *E. coli* strain IHE3034 (accession no. AM229678) using BLASTp. For each gene, a cutoff value was set based on the alignment expected value (*E*-value < 1e^−5^), sequence coverage (coverage > 80%), and identity (identity > 80%).

## RESULTS

### *In silico* analysis of the *pks* island distribution

**Fig 1:**
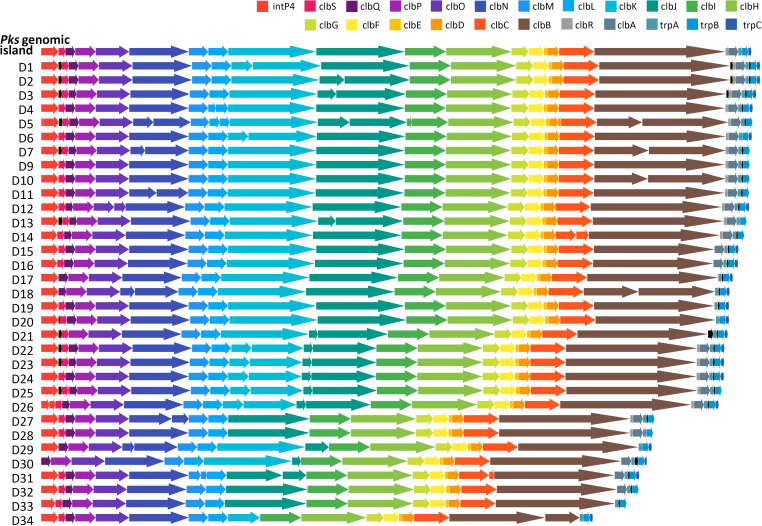
Distribution of the colibactin genome island on the *psk+* strains. Reference island corresponds to *Escherichia coli* strain IHE3034. Black arrows correspond to genes not belonging to the *pks* island.

We obtained 6,212 *E. coli* human isolates from the PATRIC database to study the distribution and structure of the *pks* island. After screening, we detected the *pks* island in 1,226 of them. However, not all harbored all the genes present in the reference *pks* island described for strain IHE3034 (Table S1). The *intP4*, *clbS*, *clbQ*, *clbK*, *clbJ*, *trpA*, and *trpB* genes were detected with a percentage of distribution between 97% and 99%, while for the *clbR*, *clbA*, and *trpC* genes their percentage was lower, between 70% and 78%, with *clbR* being the gene detected in only 70% of the strains. In addition to the fact that not all the genes are present in most of the distributions of the island, there are genes not belonging to the island distributed in the inner part of the cluster (Fig. 1).

### Selection of the *pks*-specific peptides

We further screened the 6,212 *E. coli* strains to select *pks*-specific peptides for antibody generation. We split the proteins of the colibactin genomic island of the IHE3034 strain into peptides of 20 amino acids and mapped them against the 6,212 *E. coli* strains (see Materials and methods). We selected the four peptides with the biggest detection score on the *pks*+ strains and the lowest detection score on the *pks*− strains (Table S2). These 20 amino acid length peptides were predicted on three different proteins of the *pks* cluster, that is, clbH (NRPS), clbC (PKS), and clbB (NRPS/PKS).

We further screened the four *pks*-specific peptides against the NCBI RefSeq database (44) to assess the specificity of the selected peptides in other bacterial species (see Materials and methods). With the exception of peptide #3, the other peptides appear to be specific from the colibactin-producing machinery, primarily showing significant hits against species from the Enterobacteriaceae family previously reported carrying the *pks* genomic island such as *Klebsiella*, *Citrobacter*, or *Serratia* spp. (Table S3).

### Selection of the best labeling conditions

**Fig 2:**
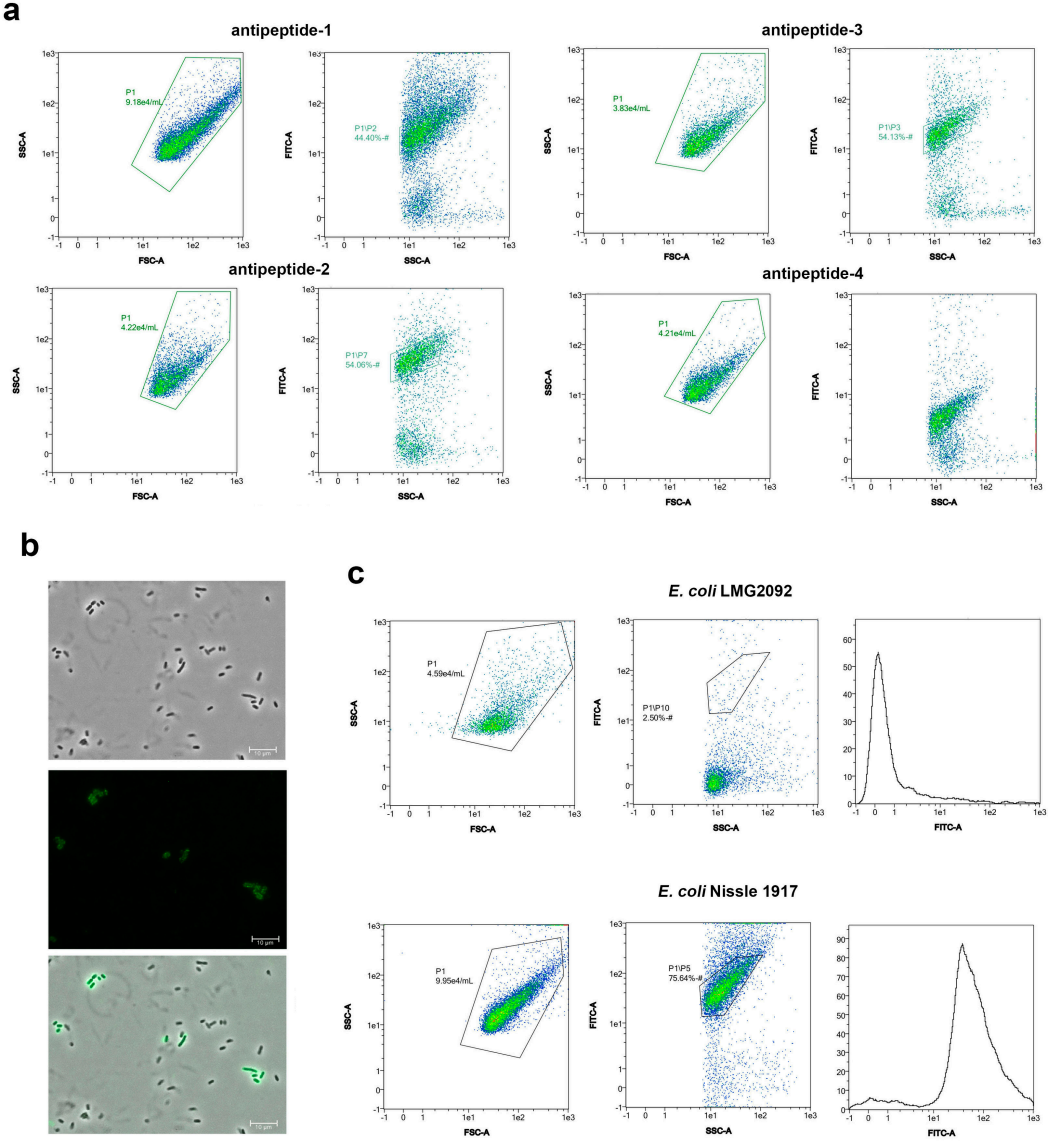
Labeling results obtained using antipeptide polyclonal antibodies against proteins from the *pks* cluster. (a) Scatter plots of representative flow cytometry (FC) experiments. Diagrams show the acquisition of marked *Escherichia coli* Nissle 1917 cell suspension in the exponential phase of growth and aeration condition with four different polyclonal antibodies. (b) Immunofluorescence microscopy photography shows the binding of FITC-antipeptide 2 antibody to the Nissle 1917 strain. (c) Labeling results obtained in nutrient broth at OD_600_ of 1.00 with antibody antipeptide 2. Dispersion diagrams and histograms show the FC acquisition of LMG2092 and Nissle 1917 strains.

We evaluated the labeling of a *pks*+ (*E. coli* Nissle 1917) and a *pks*− (*E. coli* LMG2092) strains with antipeptide 1, 2, 3, and 4 polyclonal antibodies by FC. FC analyses showed the capability of the antibodies against three of these peptides to detect the *pks+* strains, labeling approximately a 50% of the *E. coli pks*+ cells analyzed (antibodies against peptide #4 were less effective and, therefore, it was discarded for the following experiments; Fig. 2a). We also evaluated the recognition of the *pks*+ strain with polyclonal antipeptide 1, 2, and 3 antibodies conjugated with FITC using a confocal laser scanning microscope (Fig. 2b, Fig. S1).

In an attempt to improve these results, different growth conditions and optical density were taken into account to discover their influence in the expression of the *pks* machinery (Table S4). Best results (labeling ~76% of the *pks+* and ~1% of *pks*− cells) were obtained using the antibody directed to the peptide #2 over cells grown on Nutrient Broth medium at optical density of 1 OD_600_. Negative controls, using the preimmune serum conjugated with APC, further validate the specificity of the selected antibody (Fig. S2). The higher emission of *E. coli* Nissle and the lower emission of the *E. coli* LMG2092 can be deduced by comparing the scatter and histogram plots (Fig. 2c).

### Detection and isolation of *pks*+ bacteria from human gut microbiotas

Four stool samples from healthy subjects (V-HD1 to 4) were collected to validate the suitability of the proposed method to detect, isolate and eventually deplete *pks*+ cells from a complex bacteria community. This method is based on the recognition of a specific molecule on the surface of *pks*+ bacteria. In this case, the chosen target was a peptide loop inside one of the proteins encoded in the *pks* operon. Polyclonal antibodies developed against these peptides allowed to obtain, after an immunomagnetic step, two bacteria fractions, denominated positive and negative. Positive fractions contained bacteria retained by the antibodies, which were expected to be enriched in *pks*+ bacteria. On the contrary, negative fractions contained the rest of microorganisms with an expected depletion of *pks*+ bacteria. We applied this procedure on the microbiotas extracted from the four stool samples, supplemented with a proportion of 0.1%, 1%, and 10% of *E. coli* Nissle 1917 cells. Then, we assessed the *pks*+ presence in the positive and negative fractions by qualitative PCR (Fig. S3; see Materials and methods). The results showed that it is possible to deplete a portion of the *E. coli pks+* cell population from a human gut microbiota, but only when they are present above a certain abundance. In fact, with 0.1% and 1% supplementation, it was possible to detect *pks*+ cells only in the positive fraction of one donor (V-HD1), while in all the positive fractions supplemented with a proportion above 10% a positive detection was observed. However, *pks*+ cells were always detected in all the negative fractions, independently of the enrichment proportion.

In order to test whether repeated immunomagnetic steps could improve the depletion results, we repeated the experiment using a synthetic community containing both a *pks*+ (*E. coli* Nissle 1917) and a *pks*− (*E. coli* LMG2092) strain, and measured the proportion of *pks*+ cells in the positive and negative fractions after one, three, and six steps using FC. According to the number of passes, the amount of *pks*+ cells detected in the negative fraction decreased from 0.59% after the first step to 0.29% after six steps (Fig. S4).

**Fig 3:**
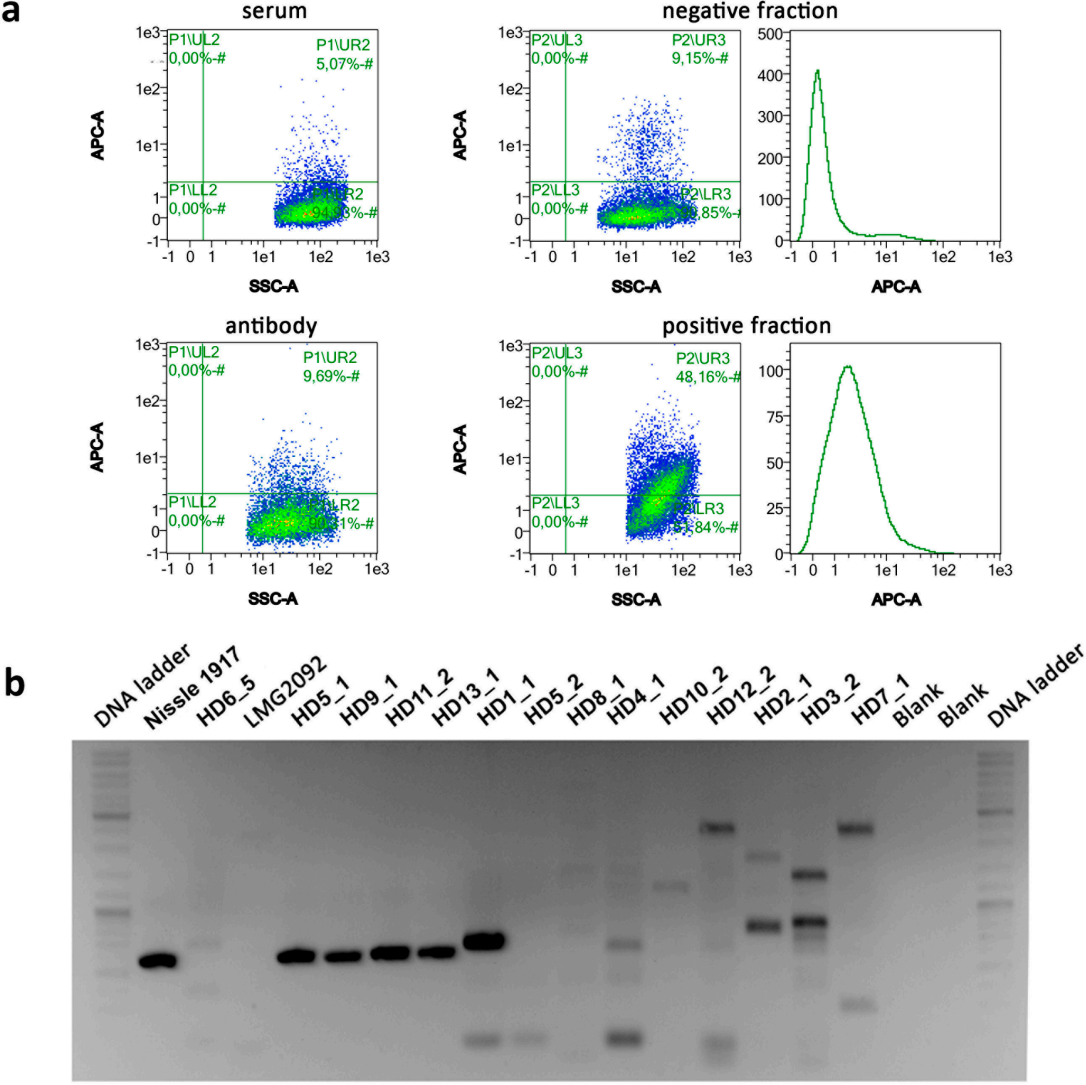
Detection, enrichment and depletion of *Escherichia coli pks*+ from gut microbiotas. (a) Scatter plots and histograms showing the flow cytometry acquisition using the antipeptides 2 polyclonal antibodies on the microbiota from a healthy donor (HD13). The microbiota labeled with the immune serum (top left), as isotype control and labeled with the antipeptide 2 antibody (bottom left), and the negative (top right) and positive (bottom right) fractions are shown. (b) Qualitative PCR of the *clbB* gene on 14 DNA samples from isolates of positive fractions from different microbiota. *E. coli* Nissle 1917 and LMG2092 strains were selected as positive and negative controls, respectively.

After validating the suitability of the methodology, the same stool samples (HD1 to 4; in this case without any *pks*+ supplementation) together with additional nine stool samples from healthy donors (HD5 to 13), were processed to detect (Fig. 3a, Fig. S5) and deplete from *pks*+ strains. After depletion, the positive fractions from these 13 samples were cultivated to validate the viability of the depleted cells. From these cultures, 39 isolates were retrieved, their 16S region was sequenced, and subsequently validated of the *clbB* gene by PCR analysis (Fig. 3b). Moreover, the complete genome of 14 of these 39 isolates was sequenced, assembled, taxonomically classified, and analyzed to detect the *pks* island (Table 1, Table S5). The results obtained with the PCR detection of the *pks* cluster were in agreement with the results obtained by analyzing the assembled genomes. Four out of the 14 analyzed isolates were detected as *pks*+ bacteria, all of them belonging to the phylogroup B2 of the *E. coli* species (Table 1, Table S5).

**TABLE 1:** Taxonomic identification, phylogenetic group, and PCR and WGS validation of 14 isolates from the positive fraction of the gut microbiotas^*a*^

Isolate	Taxonomy	Phylogroup	PCR validation	WGS validation
HD1_1	*Escherichia coli*	F	Negative	Negative
HD2_1	*Klebsiella pneumoniae*	N/A^*b*^	Negative	Negative
HD3_2	*Escherichia coli*	D	Negative	Negative
HD4_1	*Escherichia coli*	A	Negative	Negative
HD5_1	*Escherichia coli*	B2	Positive	Positive
HD5_2	*Enterococcus faecium*	N/A^*b*^	Negative	Negative
HD6_5	*Escherichia coli*	B1	Negative	Negative
HD7_1	*Citrobacter freundii*	N/A^*b*^	Negative	Negative
HD8_1	*Escherichia coli*	D	Negative	Negative
HD9_1	*Escherichia coli*	B2	Positive	Positive
HD10_2	*Enterobacter cloacae*	N/A^*b*^	Negative	Negative
HD11_2	*Escherichia coli*	B2	Positive	Positive
HD12_2	*Escherichia coli*	B2	Negative	Negative
HD13_1	*Escherichia coli*	B2	Positive	Positive

^*a*^ PCR validation was performed amplifying a region of the clbB gene.

^*b*^ N/A, not applicable.

## DISCUSSION

The human gut microbiome has been hypothesized to play a crucial role in the development and progression of CRC (27, 55 - 57). Among the different microorganisms potentially involved, the *E. coli* strains carrying the *pks* genomic island were shown to be capable of in producing genomic aberrations on colon epithelial cells (58), colon tumorigenesis in mice models of chronic inflammation and colorectal cancer (23, 59), and their presence seems to be directly related to a distinct mutational signature in patients suffering CRC (34). Acknowledging this relation and therefore the carcinogenic potential of these strains, identification, isolation, and depletion of these bacteria could help to understand the role of these strains not only in the context of CRC, but also in other diseases or physiological, metabolic, and immune processes.

Previous studies determined which strains were colibactin producers based on the detection of the *clbB* gene by PCR (25). However, to our knowledge, studies using antibodies against specific peptides of the *pks* island have never been designed. For this reason, we selected four *pks*-exclusive peptides from genes presented in all the *pks* distributions to generate polyclonal antibodies against them, and different growth conditions and optical densities were tested to determine their influence in the expression of the *pks* machinery.

These results showed that our methodology was able to detect *pks*+ strains in simple bacteria mixes. The next step was to assess whether it was also suitable for detecting, isolating, and depleting *pks*+ bacteria in human gut microbiotas. Therefore, we enriched four human gut microbiotas obtained from healthy donors with a proportion of 0.1%, 1%, and 10% of *E. coli* Nissle 1917 cells, and positive/negative fractions were obtained using our immunomagnetic protocol. In general, presence of *pks*+ bacteria was only detected in positive fractions obtained from gut microbiotas supplemented with 10% *pks*+ bacteria. Further investigation is therefore necessary to establish the appropriate conditions for optimized labeling of colibactin-producing bacteria in complex microbial communities.

In contrast to methods based on probes, our methodology allowed identification and certain level of depletion of *pks*+ bacterial strains. Further culture of depleted *pks*+ bacteria and *t* positive fractions from 13 gut microbiotas from healthy donors, allowed isolation of 39 potential *pks*+ isolates belonging to the Enterobacteriaceae family, for example, *Escherichia*, *Klebsiella*, *Enterobacter*, and *Citrobacter* genus. However, after PCR and WGS validation, we discovered that not all the bacteria retrieved harbored the *pks* island, and only 4 out of 14 isolates were detected as *pks*+ and were identified as belonging to *E. coli* species. The depletion of *pks*− bacteria from other not yet described potential *pks+* genus, such as *Enterococcus* and *Serratia*, may be related to the polyclonal nature of the antibody, raised in an animal that contains Firmicutes and y-Proteobacteria as part of the normal microbiota.

### Conclusions

To our knowledge, this represents the first method for the detection and depletion of *pks*+ bacteria in human gut microbiotas based on *in silico* predicted *pks*-specific anti-peptide antibodies. In contrast to methods based on probes, our methodology allows the depletion of low-abundance bacterial strains maintaining the viability of both targeted and non-targeted fractions of the microbiota, allowing the study of the contribution of these *pks*-carrying strains to different diseases, such as CRC, and their role in other physiological, metabolic, or immune processes.

The methodology presented in this work allows detection and isolation of *pks*+ bacteria using specific antibodies targeting extracellular loops of the *pks* machinery. However, care should be taken with this experimental approach as it would require further developments in order to obtain complete colibactin-producing bacteria depletion, as cells with no or low level of *pks*+ gene expression are not completely eliminated. Future developments of this methodology, such as implementation of fluorescence-activated cell sorting and monoclonal antibodies with high affinity will render a more advanced and efficient protocol that, coupled with other methodologies such as microbiota transplant, might be capable of modifying the gut microbiota in an animal model after modified-microbiota reimplantation.

## SUPPLEMENTAL MATERIAL

TABLE S1 msystems.00079-23-s0001.xlsx Accession numbers, classification and *pks* island distribution of the analyzed *E. coli* strains.

TABLE S2 msystems.00079-23-s0002.xlsx Selected peptides for the antibody generation.

TABLE S3 msystems.00079-23-s0003.xlsx Screening of the selected peptides against the NCBI RefSeq database. Hits against *E. coli* isolates are not reported.

TABLE S4 msystems.00079-23-s0004.xlsx Summary data on the percentage of bacteria labeled with different mediums, antibodies and optical density. The data show the mean and the standard deviation.

TABLE S5 msystems.00079-23-s0005.xlsx Summary table of the isolates obtained and the DNA concentration for the PCR experiments.

FIG S1 msystems.00079-23-s0006.tif Immunofluorescence microscopy photography showing the binding of the FITC‐anti peptide 1 (a) and 3 (b) antibodies to *E. coli* Nissle 1917.

FIG S2 msystems.00079-23-s0007.tif Labeling results obtained using antipeptide polyclonal antibodies and the preimmune serum on *E. coli* LMG2092 and Nissle 1917. (a) Scatter plots and histograms showing the flow cytometry acquisition using antipeptide #2 polyclonal antibodies on *E. coli* LMG2092 cells. (b) Scatter plots and histograms showing the flow cytometry acquisition using the preimmune serum on *E. coli* LMG2092 cells. (c) Scatter plots and histograms showing the flow cytometry acquisition using antipeptide #2 polyclonal antibodies on *E. coli* Nissle 1917 cells. (d) Scatter plots and histograms showing the flow cytometry acquisition using preimmune serum on *E. coli* Nissle 1917 cells.

FIG S3 msystems.00079-23-s0008.tif Qualitative PCR of four microbiotas (V‐HD1 to 4) supplemented with 0.1%, 1%, and 10% of *E. coli* Nissle cells. C+: positive control; MT: microbiota without supplement; F+: positive fraction; F−: negative fraction. *E. coli* Nissle 1917 was selected as the positive control.

FIG S4 msystems.00079-23-s0009.tif Improvement of the depletion results after repetitive immunomagnetic steps. (a) Scatter plots and histograms showing the flow cytometry acquisition using the antipeptides 2 polyclonal antibodies on the synthetic community containing both *E. coli* LMG2092 and Nissle 1917 cells. (b–d) Scatter plots and histograms showing the depletion results after (b) one, (c) three, and (d) six immunomagnetic steps. The negative (right) and positive (left) fractions are shown

FIG S5 msystems.00079-23-s0010.pdf Scatter plots and histograms showing the flow cytometry acquisition using the anti‐peptides 2 polyclonal antibody on 13 microbiotas from healthy donors (HD1 to 13).

ASM does not own the copyrights to Supplemental Material that may be linked to, or accessed through, an article. The authors have granted ASM a non-exclusive, world-wide license to publish the Supplemental Material files. Please contact the corresponding author directly for reuse.
