# Supplementary figures and images for: Targeted depletion of pks+ bacteria from a fecal microbiota using specific antibodies
Source: mSystems. 2023 May 23;8(3):e00079-23. doi: 10.1128/msystems.00079-23 (PMC10308883; doi:10.1128/msystems.00079-23)

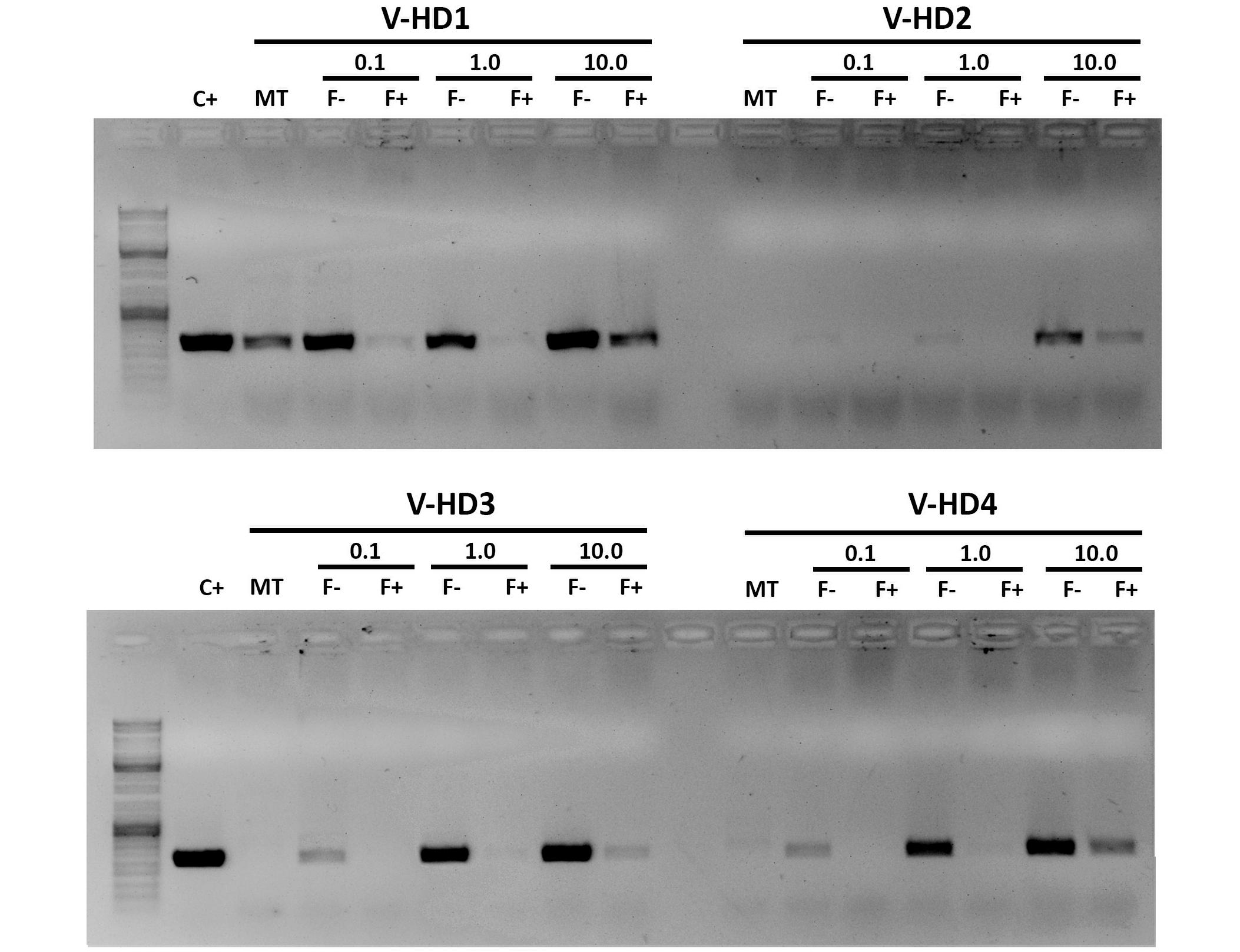

Supplement: FIG S3 — Qualitative PCR of four microbiotas (V‐HD1 to 4) supplemented with 0.1%, 1%, and 10% of E. coli Nissle cells. C+: positive control; MT: microbiota without supplement; F+: positive fraction; F−: negative fraction. E. coli Nissle 1917 was selected as the positive control. [file msystems.00079-23-s0008.tif]
