# Supplementary material for: Targeted depletion of pks+ bacteria from a fecal microbiota using specific antibodies
Source: mSystems. 2023 May 23;8(3):e00079-23. doi: 10.1128/msystems.00079-23 (PMC10308883; doi:10.1128/msystems.00079-23)
Supplement: FIG S5 — Scatter plots and histograms showing the flow cytometry acquisition using the anti‐peptides 2 polyclonal antibody on 13 microbiotas from healthy donors (HD1 to 13). [file msystems.00079-23-s0010.pdf]

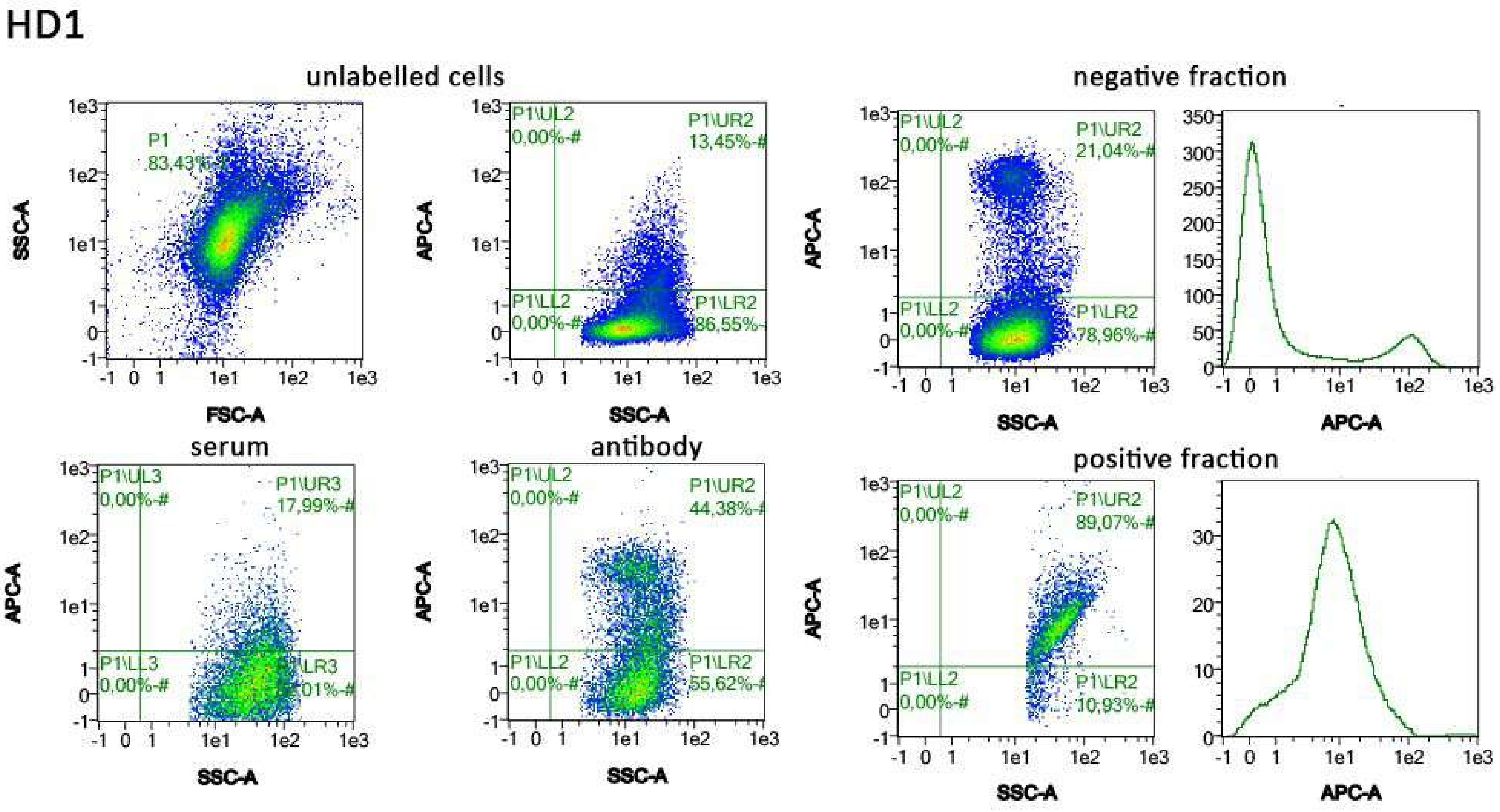
HD1
unlabelled cells
negative fraction
serum
antibody
positive fraction
P1
83,43%
P1\UL2
0,00%-#
P1\UR2
13,45%-#
P1\LL2
0,00%-#
P1\LR2
86,55%-
P1\UR2
21,04%-#
P1\LR2
78,96%-#
P1\UL3
0,00%-#
P1\UR3
17,99%-#
P1\LL3
0,00%-#
P1\LR3
P1\UR2
44,38%-#
P1\LR2
55,62%-#
P1\UR2
89,07%-#
P1\LR2
10,93%-#
SSC-A
FSC-A
APC-A

HD2

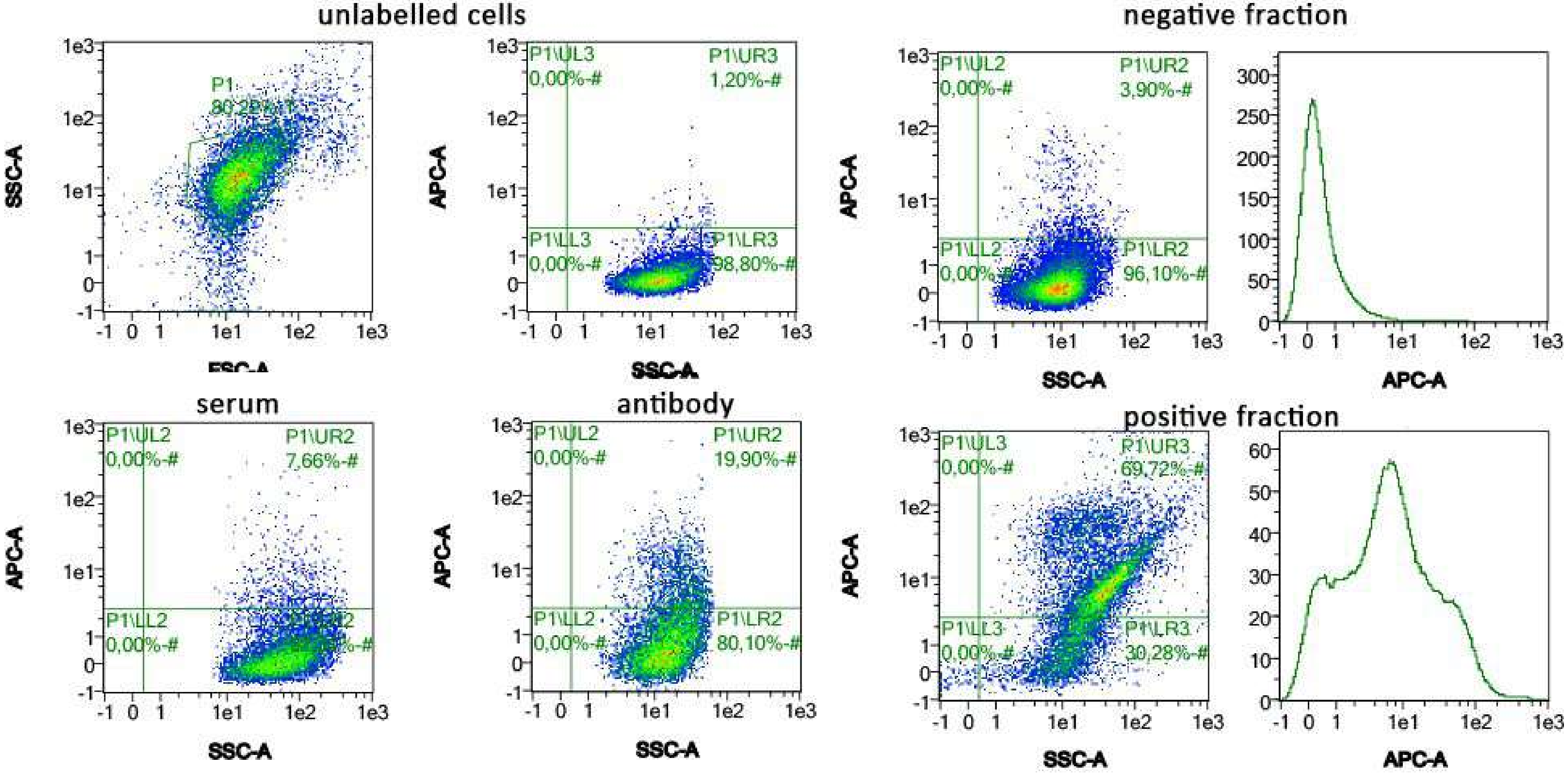
unlabelled cells
negative fraction
serum
antibody
positive fraction
P1
SSC-A
APC-A
P1\UL3
0,00%-#
P1\UR3
1,20%-#
P1\LL3
0,00%-#
P1\LR3
98,80%-#
P1\UL2
0,00%-#
P1\UR2
3,90%-#
P1\LL2
0,00%-#
P1\LR2
96,10%-#
P1\UL2
0,00%-#
P1\UR2
7,66%-#
P1\LL2
0,00%-#
P1\UL2
0,00%-#
P1\UR2
19,90%-#
P1\LL2
0,00%-#
P1\LR2
80,10%-#
P1\UL3
0,00%-#
P1\UR3
69,72%-#
P1\LL3
0,00%-#
P1\LR3
30,28%-#

HD3

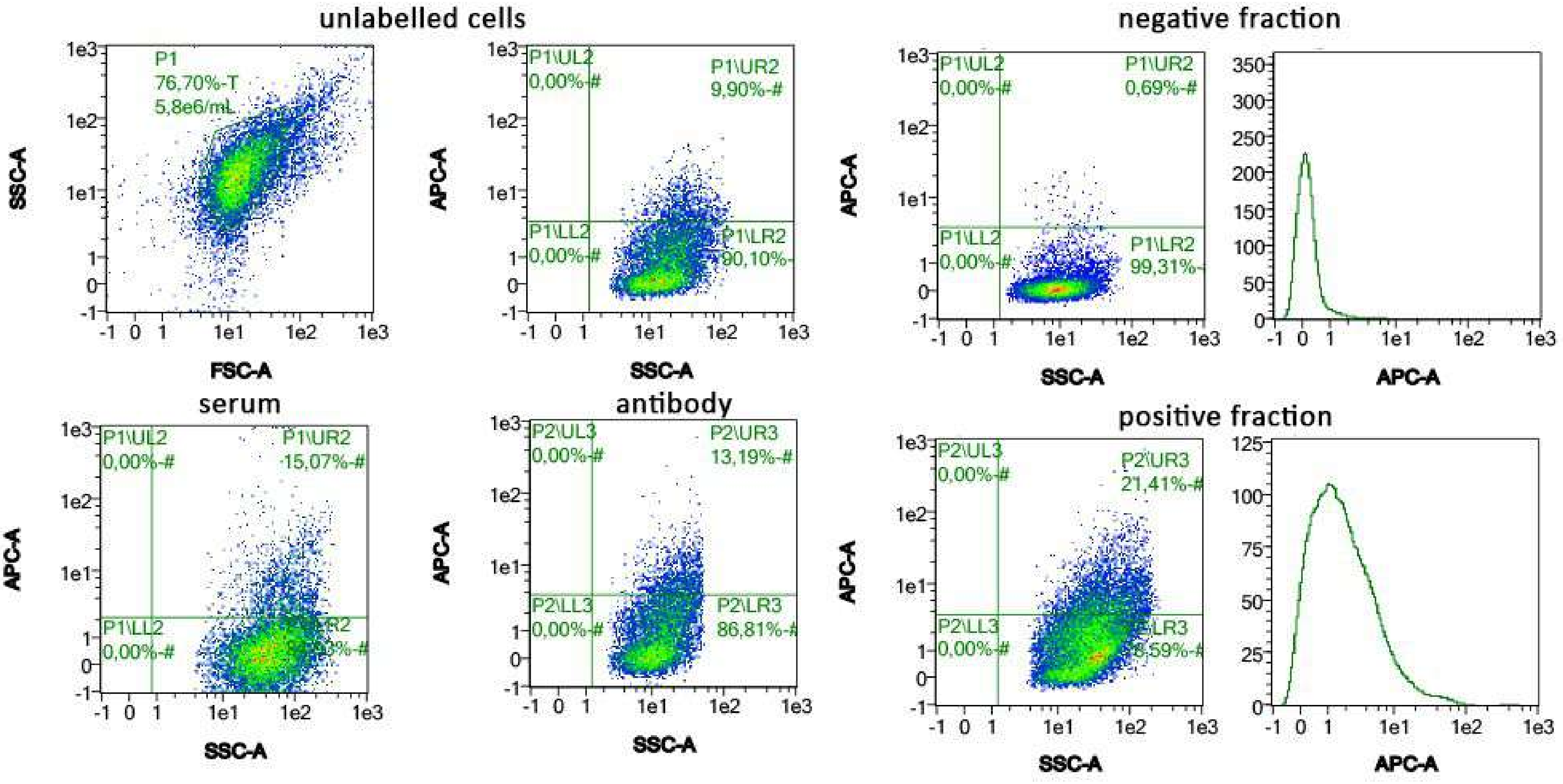
unlabelled cells
negative fraction
serum
antibody
positive fraction
P1
76,70%-T
5,8e6/mL
P1\UL2
0,00%-#
P1\UR2
9,90%-#
P1\LL2
0,00%-#
P1\LR2
90,10%-
P1\UL2
0,00%-#
P1\UR2
0,69%-#
P1\LL2
0,00%-#
P1\LR2
99,31%-
P1\UL2
0,00%-#
P1\UR2
15,07%-#
P1\LL2
0,00%-#
P2\UL3
0,00%-#
P2\UR3
13,19%-#
P2\LL3
0,00%-#
P2\LR3
86,81%-#
P2\UL3
0,00%-#
P2\UR3
21,41%-#
P2\LL3
0,00%-#
FSC-A
SSC-A
APC-A

HD4

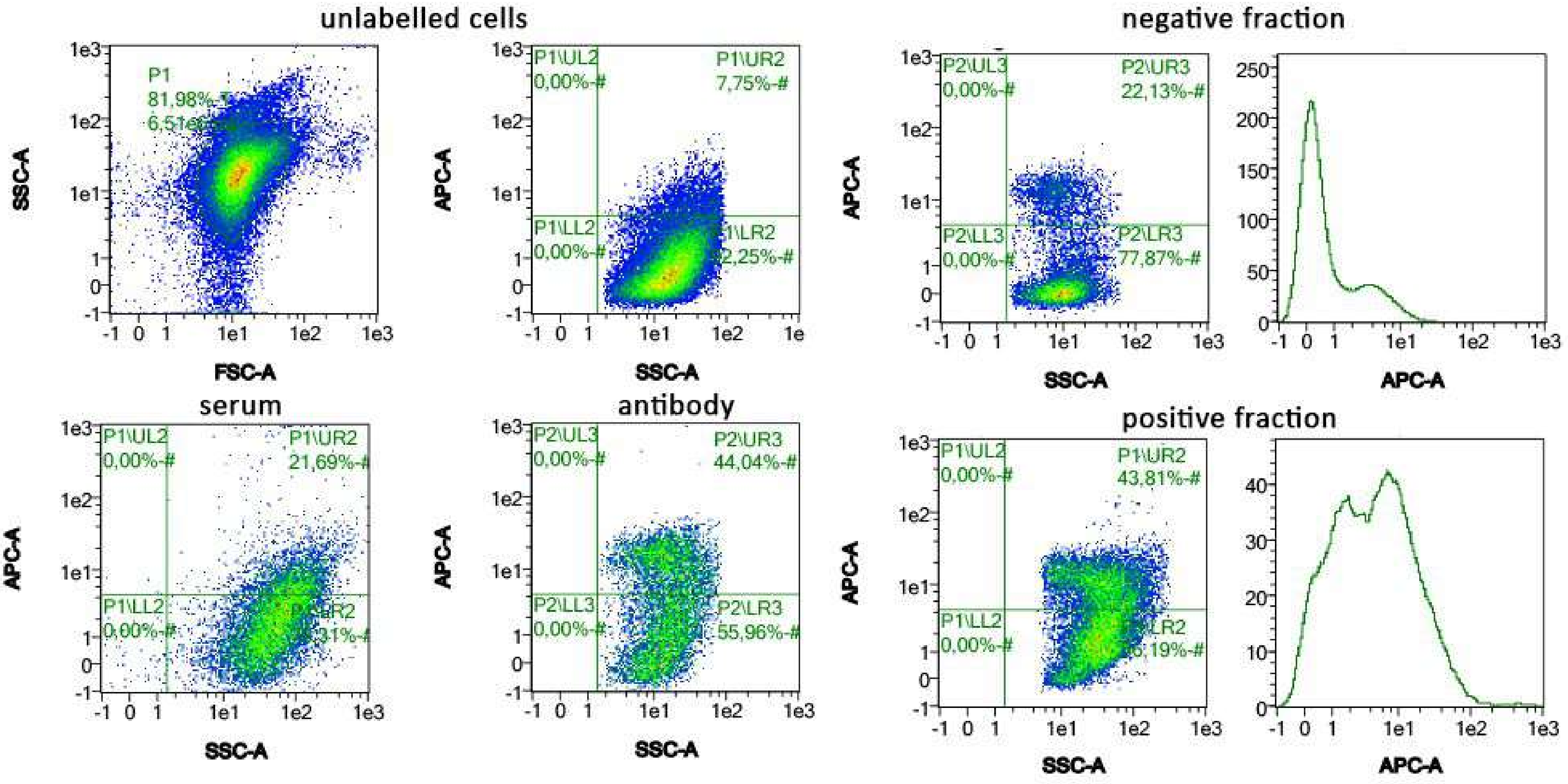

unlabelled cells
negative fraction
serum
antibody
positive fraction
P1
81,98%
P1\UL2
0,00%-#
P1\UR2
7,75%-#
P1\LL2
0,00%-#
P2\UL3
0,00%-#
P2\UR3
22,13%-#
P2\LL3
0,00%-#
P2\LR3
77,87%-#
P1\UR2
21,69%-#
P2\UR3
44,04%-#
P2\LL3
0,00%-#
P2\LR3
55,96%-#
P1\UR2
43,81%-#
SSC-A
FSC-A
APC-A

HD5

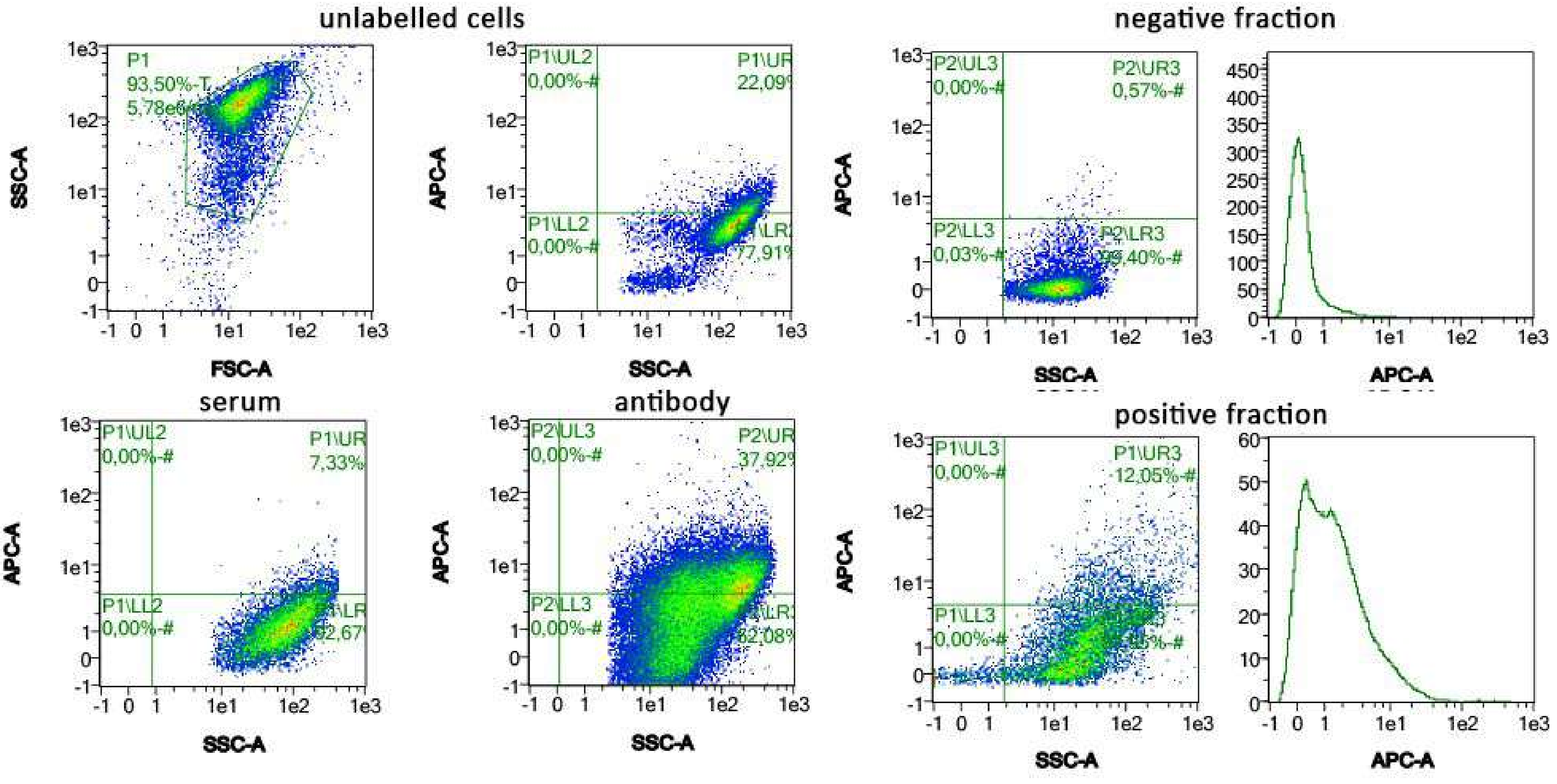
unlabelled cells
negative fraction
P1
93,50%-T
SSC-A
FSC-A
P1\UL2
0,00%-#
P1\LL2
0,00%-#
APC-A
P2\UL3
0,00%-#
P2\UR3
0,57%-#
P2\LL3
0,03%-#
serum
antibody
positive fraction
P1\UR
7,33%
P2\UR
37,92%
P1\UL3
0,00%-#
P1\UR3
12,05%-#
P1\LL3
0,00%-#

HD6

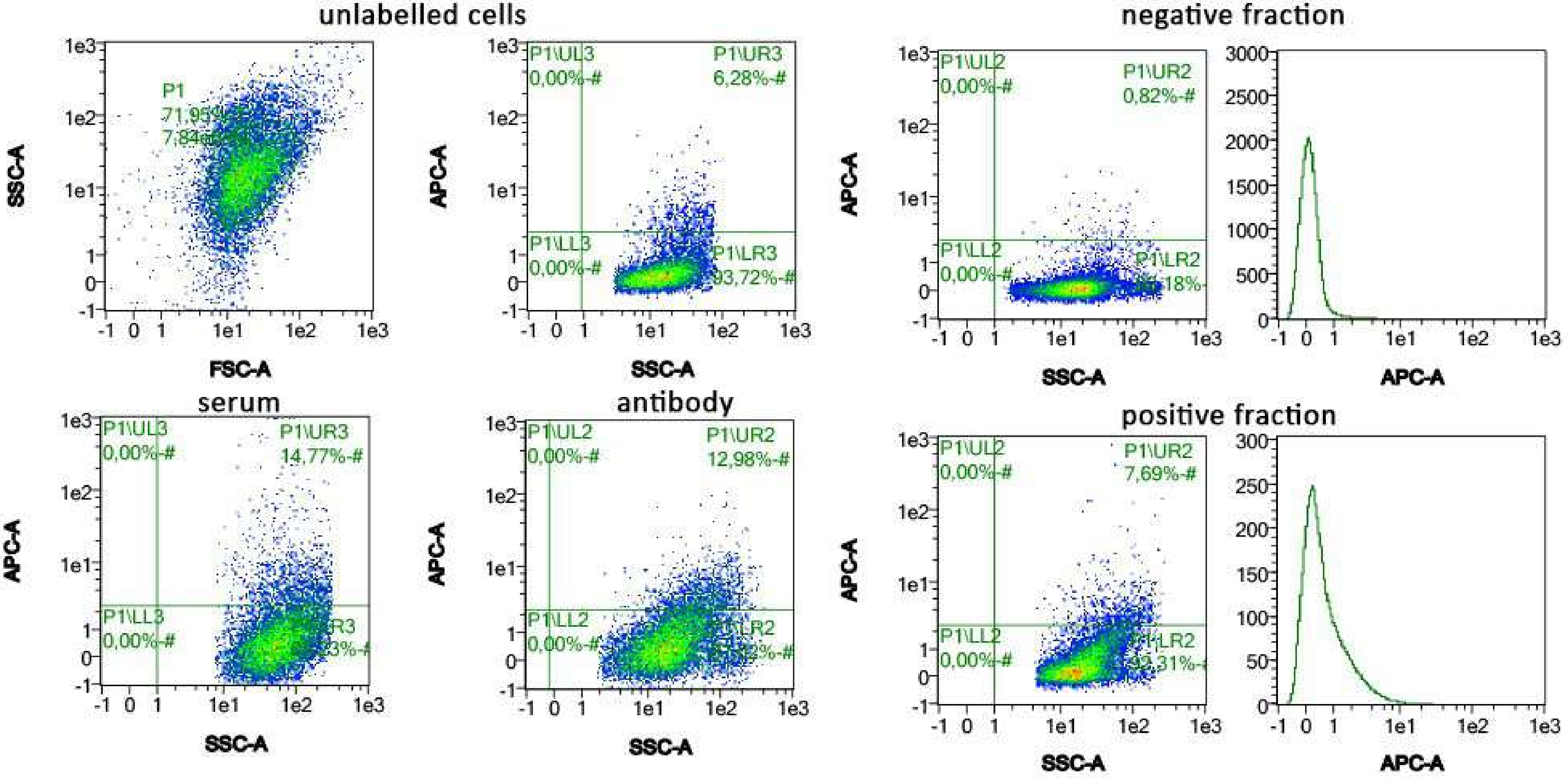

unlabelled cells
negative fraction
serum
antibody
positive fraction
P1
P1\UL3
0,00%-#
P1\UR3
6,28%-#
P1\LL3
0,00%-#
P1\LR3
93,72%-#
P1\UL2
0,00%-#
P1\UR2
0,82%-#
P1\LL2
0,00%-#
P1\LR2
P1\UL3
0,00%-#
P1\UR3
14,77%-#
P1\LL3
0,00%-#
P1\UL2
0,00%-#
P1\UR2
12,98%-#
P1\LL2
0,00%-#
P1\LR2
P1\UL2
0,00%-#
P1\UR2
7,69%-#
P1\LL2
0,00%-#
P1\LR2
92,31%-#
FSC-A
SSC-A
APC-A

HD7

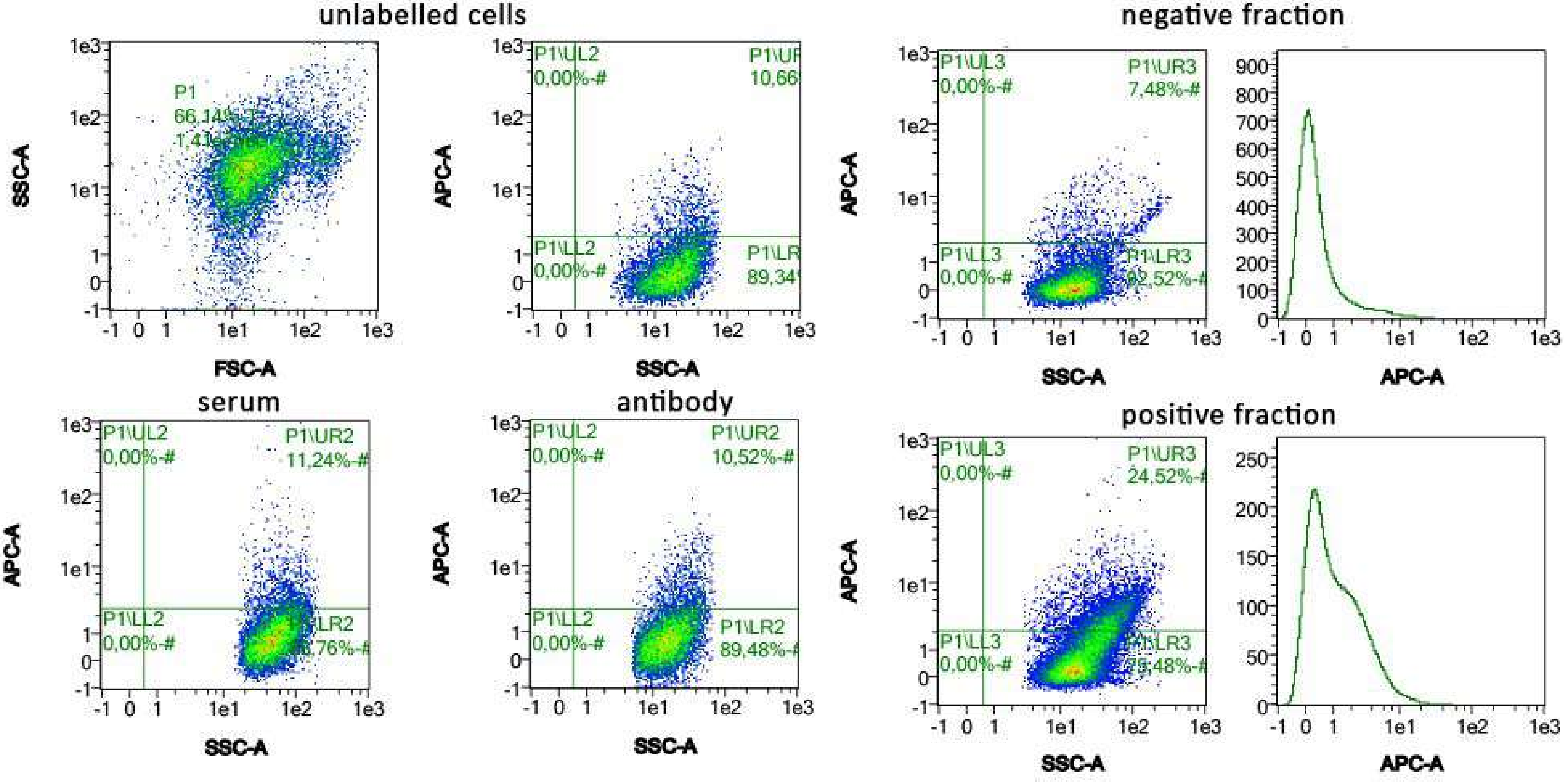
unlabelled cells
negative fraction
serum
antibody
positive fraction
P1
P1\UL2
0,00%-#
P1\LL2
0,00%-#
P1\UR2
11,24%-#
P1\UR2
10,52%-#
P1\LR2
89,48%-#
P1\UL3
0,00%-#
P1\UR3
7,48%-#
P1\LL3
0,00%-#
P1\LR3
P1\UR3
24,52%-#
FSC-A
SSC-A
APC-A

HD8

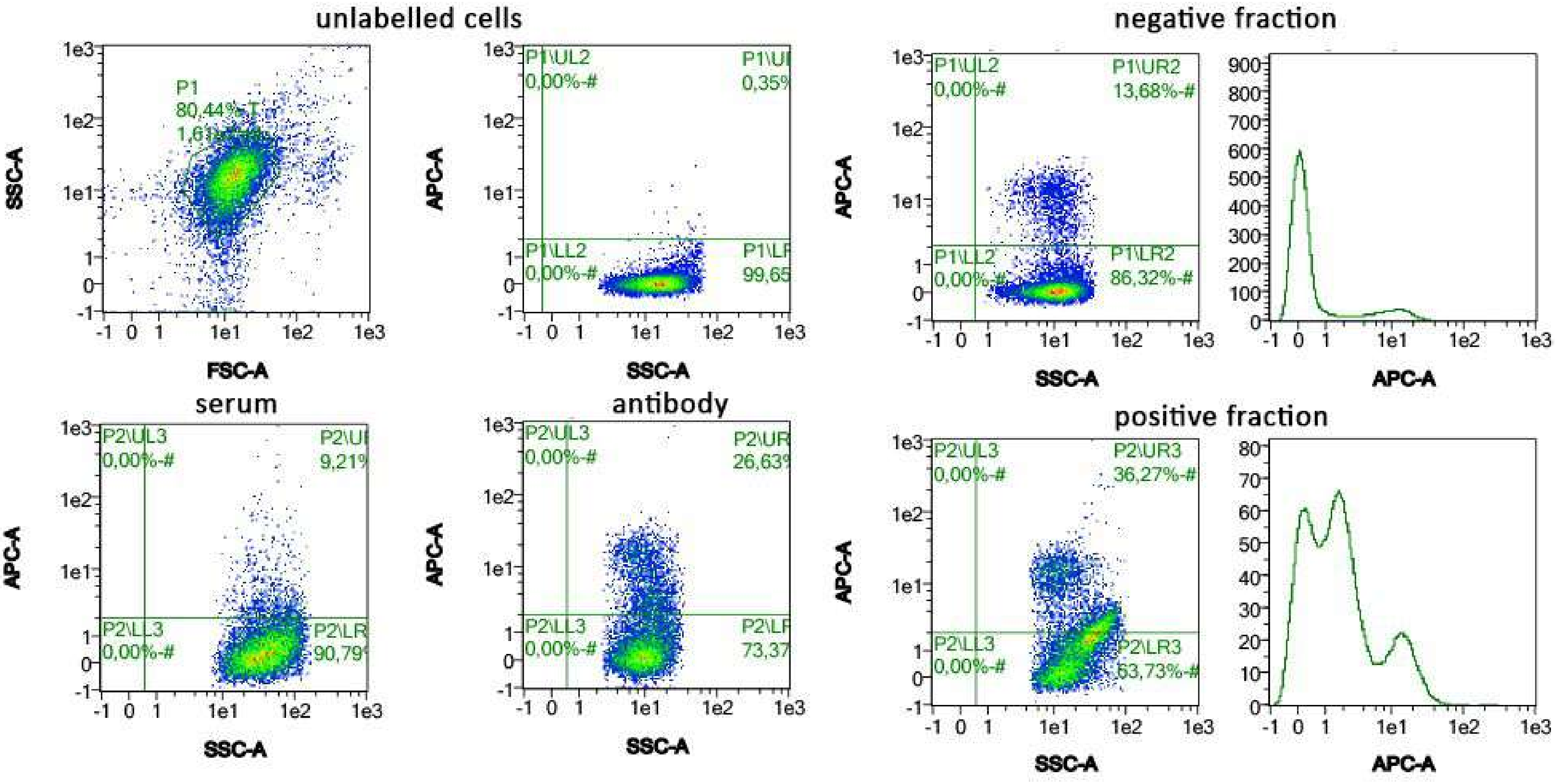

unlabelled cells
negative fraction
serum
antibody
positive fraction
P1
80,44%-T
SSC-A
FSC-A
APC-A
P1\UL2
0,00%-#
P1\LL2
0,00%-#
0,35%
99,65
P1\UR2
13,68%-#
P1\LR2
86,32%-#
P2\UL3
0,00%-#
P2\LL3
0,00%-#
9,21%
90,79%
26,63%
73,37
P2\UR3
36,27%-#
P2\LR3
63,73%-#

HD9

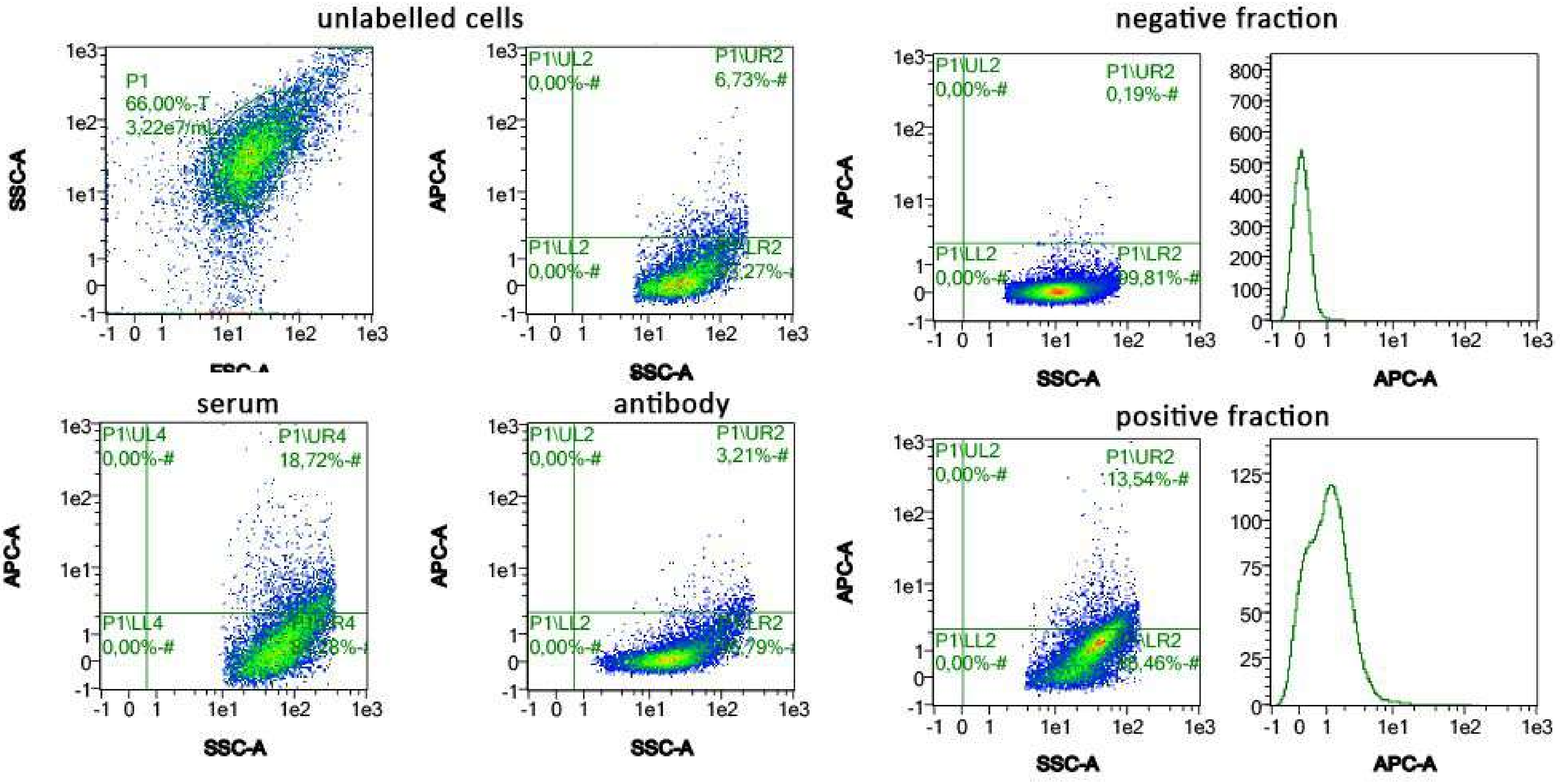
unlabelled cells
negative fraction
serum
antibody
positive fraction
P1
66,00%-T
3,22e7/mL
SSC-A
FSC-A
APC-A
P1\UL2
0,00%-#
P1\UR2
6,73%-#
P1\LL2
0,00%-#
P1\UR2
0,19%-#
P1\LR2
99,81%-#
P1\UL4
0,00%-#
P1\UR4
18,72%-#
P1\LL4
0,00%-#
P1\UR2
3,21%-#
P1\UR2
13,54%-#

HD10

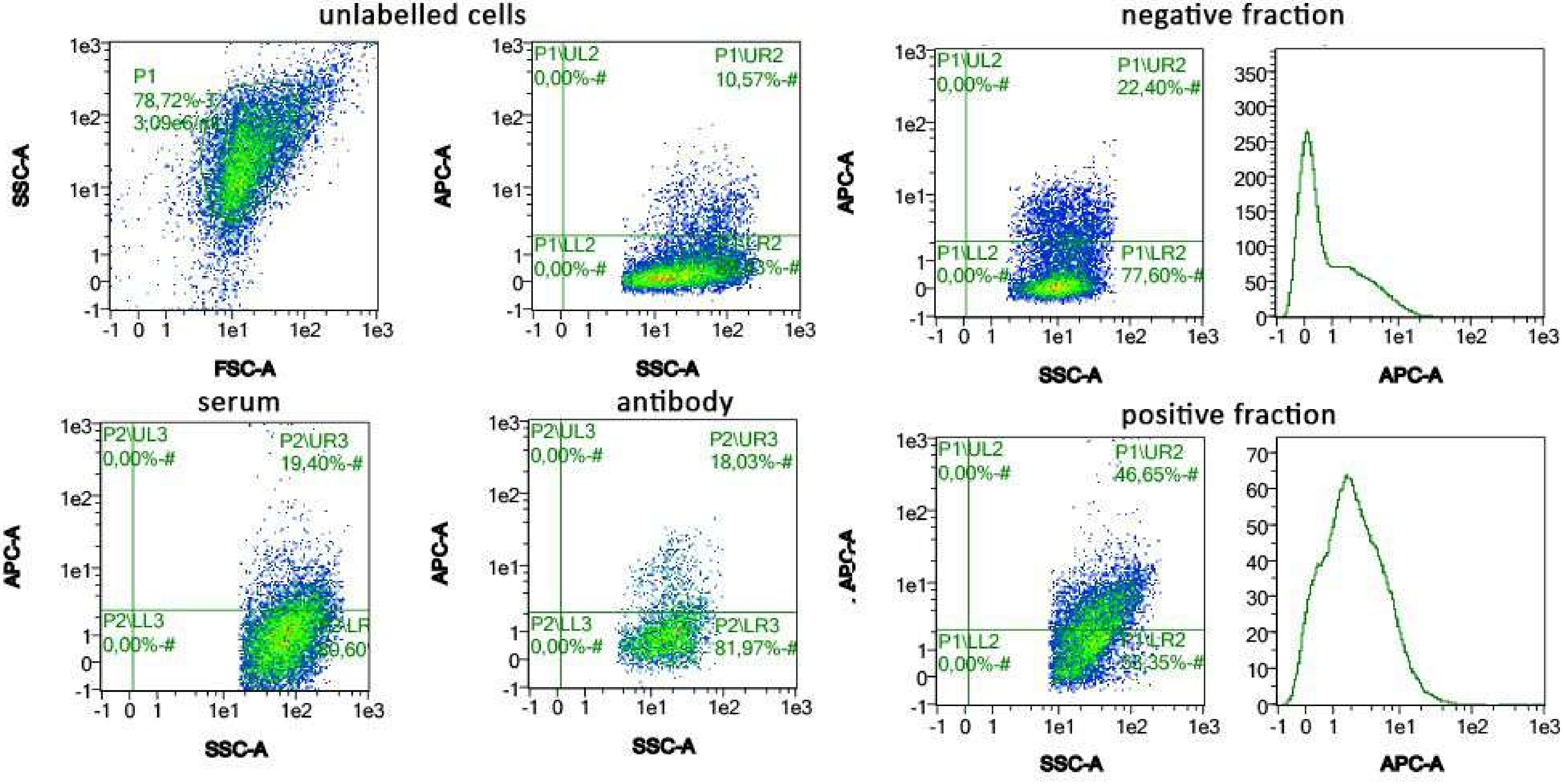
unlabelled cells
negative fraction
serum
antibody
positive fraction
P1
78,72%
P1\UL2
0,00%-#
P1\UR2
10,57%-#
P1\LL2
0,00%-#
P1\UR2
22,40%-#
P1\LR2
77,60%-#
P2\UL3
0,00%-#
P2\UR3
19,40%-#
P2\LL3
0,00%-#
P2\UR3
18,03%-#
P2\LR3
81,97%-#
P1\UR2
46,65%-#
FSC-A
SSC-A
APC-A

HD11

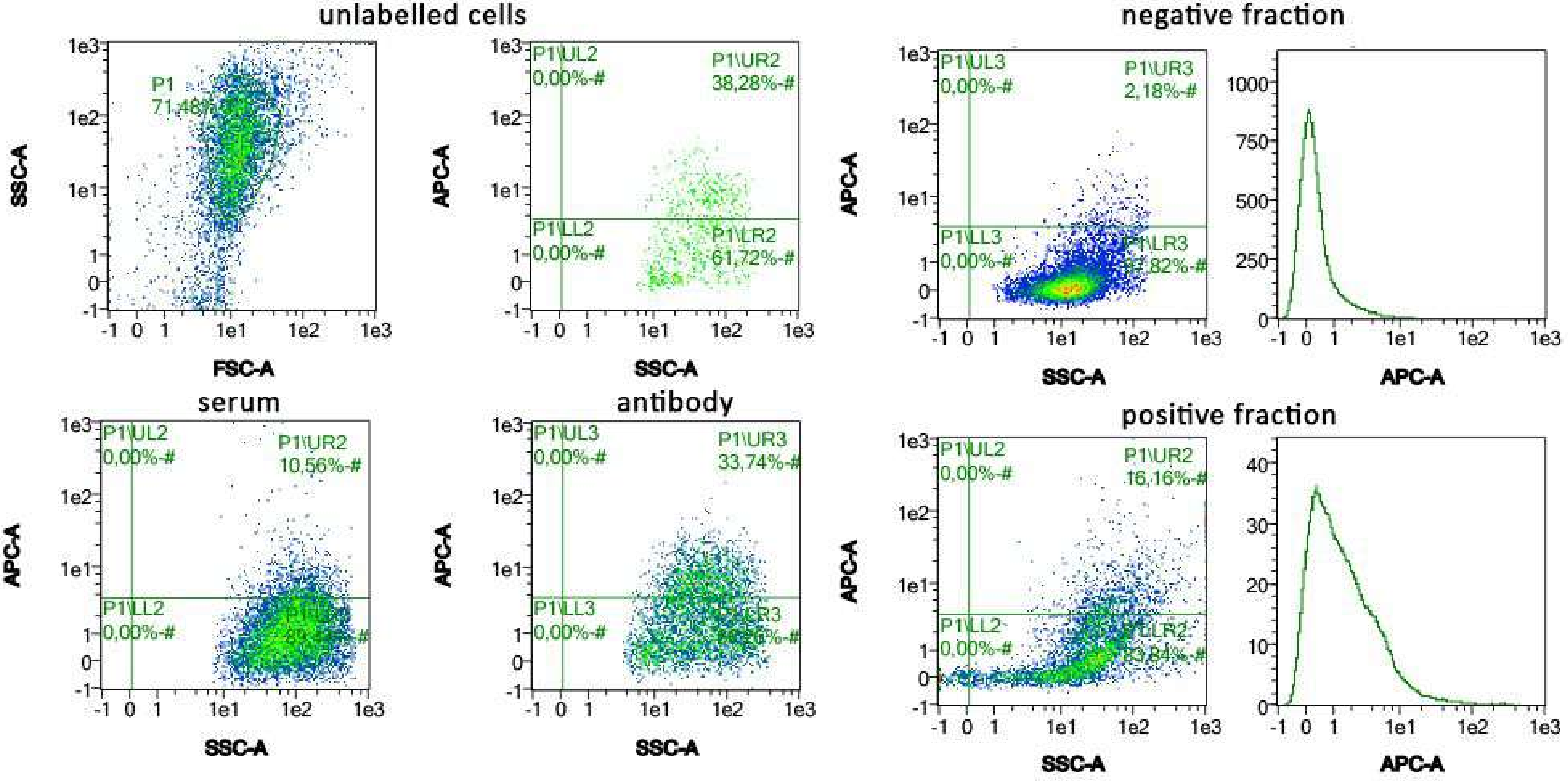

unlabelled cells
negative fraction
serum
antibody
positive fraction
P1
SSC-A
FSC-A
APC-A
P1\UL2
0,00%-#
P1\UR2
38,28%-#
P1\LL2
0,00%-#
P1\LR2
61,72%-#
P1\UL3
0,00%-#
P1\UR3
2,18%-#
P1\LL3
0,00%-#
P1\LR3
P1\UL2
0,00%-#
P1\UR2
10,56%-#
P1\LL2
0,00%-#
P1\UL3
0,00%-#
P1\UR3
33,74%-#
P1\LL3
0,00%-#
P1\UL2
0,00%-#
P1\UR2
16,16%-#
P1\LL2
0,00%-#
83,84%-#
1000
750
500
250
40
30
20
10
0
-1
1
1e1
1e2
1e3

HD12

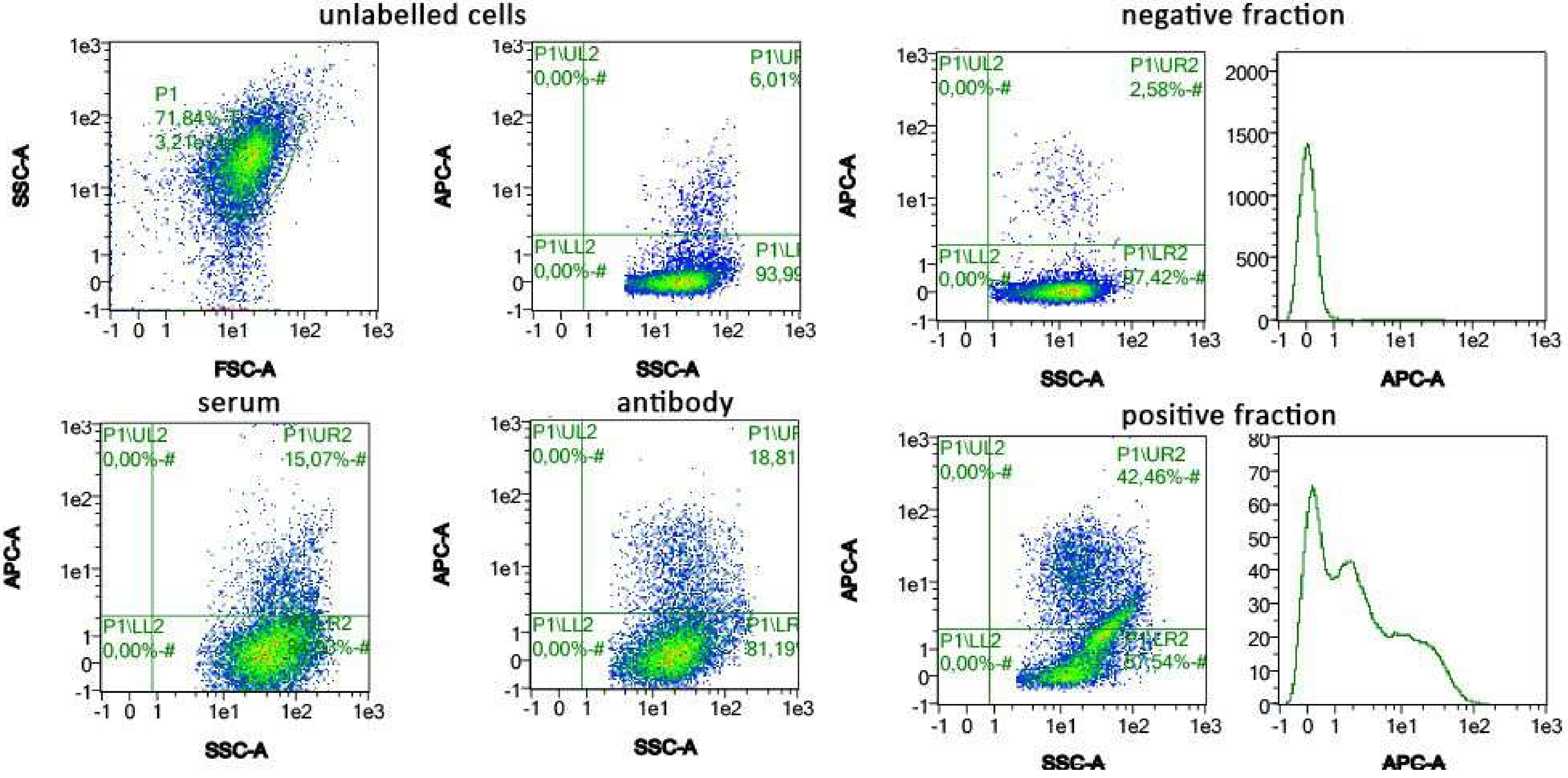

unlabelled cells
negative fraction
serum
antibody
positive fraction
P1
71,84%
SSC-A
FSC-A
APC-A
P1\UL2
0,00%-#
P1\LL2
0,00%-#
6,01%
93,99
P1\UR2
2,58%-#
P1\LR2
97,42%-#
P1\UR2
15,07%-#
18,81
81,19%
P1\UR2
42,46%-#
P1\LR2
57,54%-#

HD13

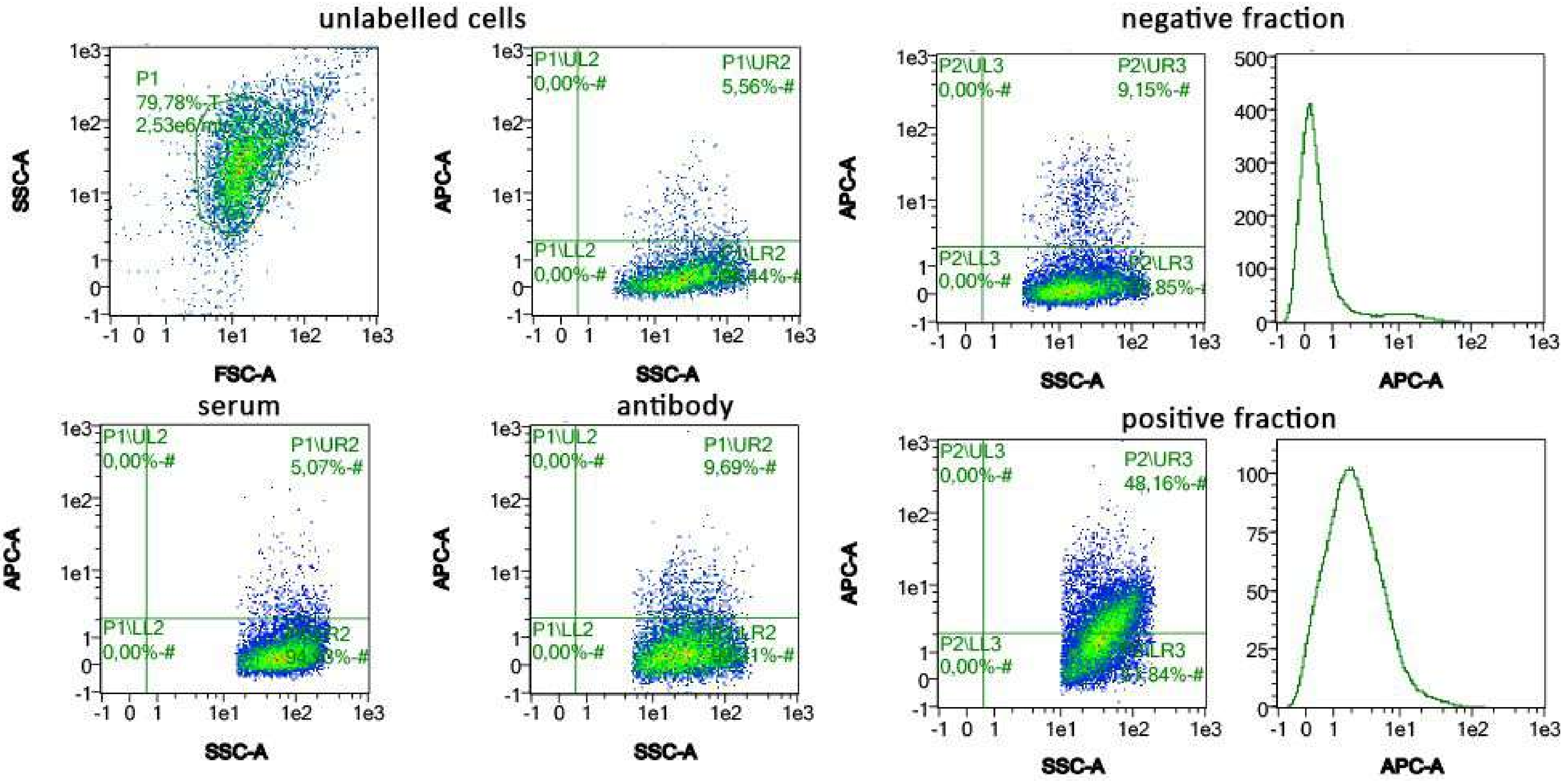
unlabelled cells
P1
79,78%-T
2,53e6/ml
SSC-A
FSC-A
P1\UL2
0,00%-#
P1\UR2
5,56%-#
P1\LL2
0,00%-#
APC-A
SSC-A
negative fraction
P2\UL3
0,00%-#
P2\UR3
9,15%-#
P2\LL3
0,00%-#
APC-A
SSC-A
APC-A
serum
P1\UL2
0,00%-#
P1\UR2
5,07%-#
P1\LL2
0,00%-#
APC-A
SSC-A
antibody
P1\UL2
0,00%-#
P1\UR2
9,69%-#
P1\LL2
0,00%-#
APC-A
SSC-A
positive fraction
P2\UL3
0,00%-#
P2\UR3
48,16%-#
P2\LL3
0,00%-#
APC-A
SSC-A
APC-A
